# Novel entry to the synthesis of (*S*)- and (*R*)-5-methoxycarbonylhydroxymethyluridines – a diastereomeric pair of wobble tRNA nucleosides†

**DOI:** 10.1039/c9ra08548c

**Published:** 2019-12-06

**Authors:** Robert Borowski, Agnieszka Dziergowska, Elzbieta Sochacka, Grazyna Leszczynska

**Affiliations:** Institute of Organic Chemistry, Lodz University of Technology Zeromskiego 116 90-924 Lodz Poland grazyna.leszczynska@p.lodz.pl +4842 636 55 30 +4842 631 31 50

## Abstract

Two novel methods for the preparation of the virtually equimolar mixtures of (*S*)- and (*R*)-diastereomers of 5-methoxycarbonylhydroxymethyluridine (mchm^5^U) have been developed. The first method involved α-hydroxylation of a 5-malonate ester derivative of uridine (5) with SeO_2_, followed by transformation to (*S*)- and (*R*)-5-carboxymethyluridines (cm^5^U, 8) and, finally, into the corresponding methyl esters. In the second approach, (*S*)- and (*R*)-mchm^5^-uridines were obtained starting from 5-formyluridine derivative (9) by hydrolysis of the imidate salt (11) prepared in the acid catalyzed reaction of 5-cyanohydrin-containing uridine (10b) with methyl alcohol. In both methods, the (*S*)- and (*R*) diastereomers of mchm^5^U were effectively separated by preparative C18 RP HPLC.

## Abstract 2

Two novel methods for the preparation of the virtually equimolar mixtures of (*S*)- and (*R*)-diastereomers of 5-methoxycarbonylhydroxymethyluridine (mchm^5^U) have been developed.

## Graphical abstract

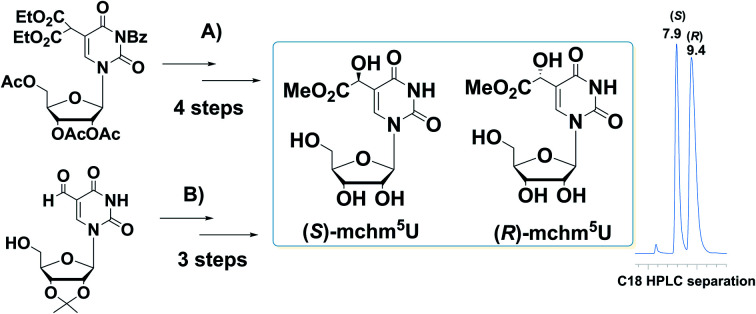

## Introduction

5-Substituted uridines constitute a class of biologically important modified nucleosides located predominantly at the wobble position 34 (the first anticodon letter) of transfer RNAs from all domains of life.^1^ Wobble uridines are essential for efficient and accurate decoding of genetic information.^2,3^ Their absence often leads to translation defects and severe diseases.^3,4^

**Fig. 1:**
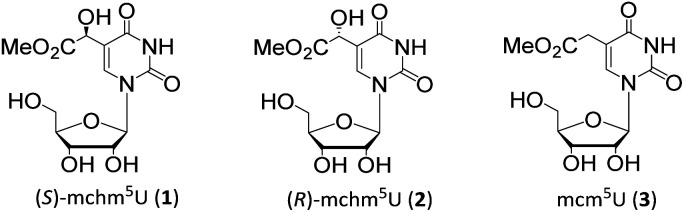
Chemical structures of (*S*)-5-methoxycarbonylhydroxymethyluridine ((*S*)-mchm^5^U, 1), (*R*)-5-methoxycarbonylhydroxymethyluridine ((*R*)-mchm^5^U, 2) and 5-methoxycarbonylmethyluridine (mcm^5^U, 3).

Wobble (*S*)- and (*R*)-5-methoxycarbonylhydroxymethyluridines ((*S*)-mchm^5^U, 1, (*R*)-mchm^5^U, 2, Fig. 1), represent a unique example of a diastereomeric pair of modified tRNA nucleosides. In mammals, the (*S*)-isomer 1 was identified in tRNAs^Gly^_UCC_ while (*R*)-mchm^5^U (2) in tRNAs^Arg^_UCG_.^5,6^ Notably, (*S*)-mchm^5^U (1) was also detected in tRNAs^Gly^UCC from insects (*e.g. B. mori*), worms (*e.g. C. elegans*) and plants (*e.g. A. thaliana*).^5,7–9^

tRNAs bearing either of these diastereomeric nucleosides are derived from the corresponding tRNAs containing 5-methoxycarbonylmethyluridine (mcm^5^U, 3, Fig. 1).^5,6^ In mammalian tRNAs, the cm^5^U_34_ → mcm^5^U_34_ methyl transfer reaction, as well as subsequent stereoselective oxidation (C–H → C–OH conversion) to (*S*)-mchm^5^U_34_, are catalyzed by an ALKBH8 enzyme,^5,6^ the member of an AlkB protein family,^10^ which are mainly involved in DNA/RNA repair processes.^5,6^ Human cells deprived of ALKBH8 were found to have reduced the endogenous level of mcm^5^U tRNA wobble modification and increased sensitivity to DNA-damaging agents.^11^ This observation indicates possible connection between a regulatory mechanism in DNA/RNA damage response pathways and modulation of tRNA modification. Interestingly, a negative correlation was discovered between the level of ALKBH8 and urothelial cancer progression.^12^ Additionally, some recessive truncating mutations in human ALKBH8 gene were recently shown to cause intellectual disability associated with the absence of (*S*)-mchm^5^U, (*R*)-mchm^5^U, m^5^U, mcm^5^Um or mcm^5^s^2^U units in total tRNA.^13^

Undoubtedly, efficient and reliable methods of synthesis of stereochemically defined nucleosides 1 and 2 would facilitate research on the biological activities of ALKBH proteins, and on the unknown path of introduction of (*R*)-mchm^5^U units to the mammalian tRNA^Arg^_UCG_. The methods reported to date are based on a regioselective C5-lithiation of the uracil residue with *n*-BuLi and a subsequent reaction of the heterobase C5-carbanion with ethyl or butyl glyoxylate (H(O)CCH_2_COOR, R = Bu, Et).^6,7,14^ Kawakami and co-workers utilized the coupling of mchm^5^-Ura with a ribose unit using the Vorbruggen's method.^7^ Next two procedures involved C5-lithiation of uridine^14^ or 5-bromouridine^6^ with the regioselectivity controlled by TBDMS protection of the nucleoside sugar moiety. The C5-lithiated species were then treated with butyl or ethyl glyoxylate to give a mixture of fully protected diastereomers, which were separated by chromatographic methods. Subsequent methanolysis of each TBDMS-protected isomer provided pure nucleosides 1 and 2. However, because of restrictive conditions of the lithiation and unavoidable partial polymerization of glyoxylate, the aforementioned protocols are rather poorly reproducible.

**Scheme 1:**
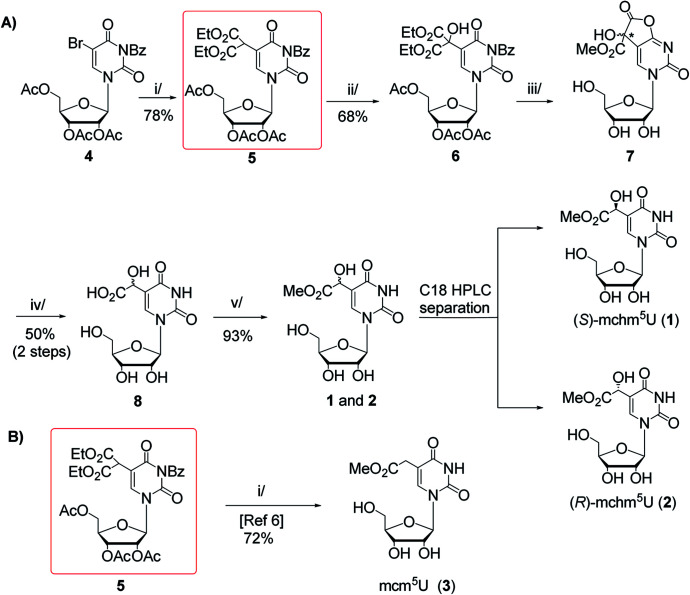
(A) Synthesis of (*S*)- and (*R*)-5-methoxycarbonylhydroxymethyluridine (1 and 2) with 5-malonylated uridine 5 as a key intermediate. Reagents and conditions: i/diethyl malonate, DBU, THF, rt, 15 h; ii/SeO_2_, dioxane, reflux, 18 h; iii/2.2 M MeONa/MeOH, MeOH, rt, 20 h; iv/TFA : H_2_O (1 : 1, v/v), 60 °C, 15 h; v/1 M HCl/MeOH, rt, 2 h; (B) Synthetic route to 5-methoxycarbonylmethyluridine (3) according to Fu *et al.*^6^ i/MeONa/MeOH, 50 °C, 16 h.

**Scheme 2:**
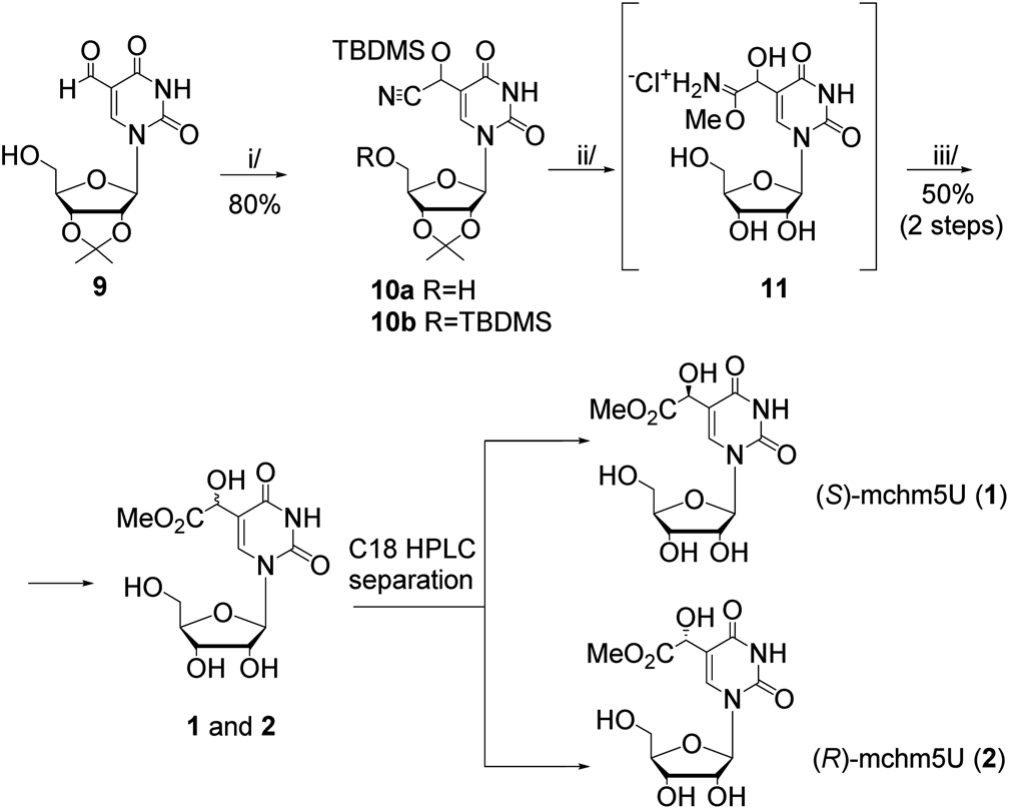
The synthesis of (*S*)- and (*R*)-5-methoxycarbonylhydroxy-methyluridine (1 and 2) starting with 5-formyluridine 9. Reagents and conditions: i/TBDMSCN, NEt_3_, CH_3_CN, rt, 2 h; ii/4 M HCl/MeOH, 5 °C, 2 h; iii/H_2_O, 5 °C, 2 h.

In this work, we present two novel methods for preparation of 1 and 2, where instead of the formation of the uridine C5-carbanion, the final modification was introduced by the transformation of nucleoside substrates containing a methyl-derived substituent at C5-uracil position (so-called C-5,1-functionalized uridines), namely 5-(diethyl malonate-yl) derivative of protected uridine 5 (Scheme 1) or 5-formyluridine derivative 9 (Scheme 2). Notably, 5-formyluridine 9 was converted to 1–2*via* a 5-cyanohydrin derivative, the synthesis and useful reactivity of which is reported for the first time.

## Results and discussion

Our first approach to the preparation of 1 and 2 was based on the assumption that the pivotal OH group in mchm^5^U can be introduced by selective SeO_2_-mediated oxidation of C-5,1 carbon in 2′,3′,5′-tri-*O*-acetyl-*N*^3^-benzoyl-5-(bis(ethoxycarbonyl)methyl)-uridine (5) (“malonate” derivative, Scheme 1A). We appreciated the accessibility of the “malonate” derivative (5), which was a substrate in synthesis of 5-methoxycarbonylmethyluridine (3) (Scheme 1B).^6^ Noteworthy, 5 could be used in preparation of all nucleosides 1–3 employing the same sequence of reactions: deprotection, decarboxylation and methyl esterification.

The “malonate” derivative 5 was obtained in a DBU promoted reaction of 2′,3′,5′-*O*-triacetyl-*N*^3^-benzoyl-5-bromouridine (4) and diethyl malonate (ESI, Fig. S1 and S2†).^6,15^ The C-5,1-oxidation upon treatment with SeO_2_ was optimized in terms of molar excess (1.5–4 equiv.), temperature (25–100 °C), solvent (1,4-dioxane, *t*-BuOH, *t*-BuOH/1,4-dioxane, CH_2_Cl_2_ in the presence of *t*-BuOOH as co-oxidant), and reaction time (5–48 h).^16^ The best yield of the hydroxyl derivative 6 was achieved using 4 equivalents of SeO_2_ in boiling 1,4-dioxane for 18 h. It was isolated by chromatography on a silica gel column in 68% yield and its structure was confirmed by NMR and MS data (ESI, Fig. S3 and S4†).

Unfortunately, our subsequent attempts at deprotection (alkaline hydrolysis of acetyl, benzoyl, and ethyl ester groups) and simultaneous decarboxylation in 6 using 2.2 M MeONa–MeOH at 50 °C (a one-pot procedure reported by Fu for a 5 → 3 conversion, Scheme 1B)^6^ led to a complex mixture of products. Thus, to perform only the deprotection, we treated 6 with 2.2 M NaOMe–MeOH at room temperature.^15^ After 20 h, virtually quantitative formation of a highly polar compound was noted, which was deprived of acetyl and benzoyl groups, but contained one methoxycarbonyl substituent (ESI, Fig. S5 and S6†). Because in the relevant ^1^H and ^13^C NMR spectra certain resonance lines were doubled (ESI, Fig. S5 and S6†) we assumed the formation of diastereomeric species. Indeed, NMR and MS data allowed to identify a cyclic intermediate 7 bearing a new stereogenic centre (marked with an asterisk), which presumably was formed in independent reactions of –COOEt → –COOMe conversion and γ-lactonization, the latter with the participation of the O4-atom. To open the lactone and to perform subsequent decarboxylation, crude 7 was treated with 50% aqueous trifluoroacetic acid (TFA) at 60 °C.^15^ After 15 h, TLC analysis showed the presence of a new product, which was even more polar than the parent 7. It was isolated by chromatography on an open column (C8-RP) in 50% yield (calculated over compound 6) and identified as a *ca.* 1 : 1 mixture of (*S*)- and (*R*)-5-carboxyhydroxymethyluridine 8 (ESI, Fig. S7–S9†). Treatment of the mixture of (*S*)- and (*R*)-acids 8 with anhydrous 1 M HCl–MeOH at room temperature for 2 h furnished the mixture of (*S*)- and (*R*)-mchm^5^U diastereomers (1–2) (ESI, Fig. S10–S13†), which was resolved onto stereochemically pure species by RP HPLC in 43% and 50% yield, respectively (a preparative C18 column was eluted with water as shown in ESI, Fig. S17A;† conditions for separation of diastereomers were established based on the analytical HPLC, see ESI, Fig. S17B†).

Our second approach to the preparation of 1 and 2 was based on the prediction that 5-formyluridine 9 can be effectively transformed to the appropriate 5-cyanohydrin derivative and then to the imidate salt by a Pinner reaction (Scheme 2).

The recent development of 5-formylpyrimidine nucleosides as epigenetic modifications significantly improved the synthetic availability of these nucleosides.^17^ In the present research, we used our earlier reported method, based on selective oxidation of the appropriately protected 5-hydroxymethyluridine with activated MnO_2_,^18^ to prepare a 5-formyluridine derivative 9 (Scheme 2). Initial experiments aimed at the conversion of the 5-formyl group into a cyanohydrin function (C5–CH(CN)OH) using KCN/acetic acid^19^ failed because several attempts at isolation of the product ended with predominant recovery of the substrate. This reversibility was debarred by the use of TBDMS-cyanide^20^ (up to 2-fold molar excess), so the hydroxyl group of the cyanohydrin function was instantly protected with a TBDMS group (–C5–CH(CN)OTBDMS). However, after 15 minutes, two products were observed regardless of the reagent ratio. The relevant NMR spectra revealed that the desired *O*-silylated cyanohydrin adduct (10a, ESI, Fig. S14†) was accompanied by its derivative 10b bearing also 5′-*O*-TBDMS group (ESI, Fig. S15 and S16†). Our attempts to increase the selectivity of the cyanohydrin protection were unsuccessful. Therefore, using 3-fold molar excess of TBDMS-CN the bis-silylated compound 10b was obtained (in 80% yield) and used for further transformations.

Hydrogen chloride catalyzed addition of methanol to the CN group in 10b (the Pinner reaction)^21,22^ led to an imidate hydrochloride salt 11 (Scheme 2), from which residual HCl was carefully removed to have the conditions safe for the methyl ester group present in the final nucleoside. Subsequent simple hydrolysis (the treatment with water at 5 °C for 2 h)^21,22^ furnished a mixture of isomers of mchm^5^U (1–2). Their identity was confirmed by HPLC comparison with the reference samples. The isomeric products were separated by RP HPLC (a preparative C18 column, water as an eluent) and each diastereomer was obtained in *ca.* 25% yield (the 50% yield obtained for both diastereomers refers to 10b).

As expected, the reagent used in the Pinner reaction (4 M HCl/MeOH, 2 h) concomitantly removed the 2′,3′-isopropylidene and TBDMS protecting groups. The use of 2 M HCl/MeOH led to longer reaction time (4 h) while 1 M HCl/MeOH resulted in the recovery of 5-formyluridine, probably because of the preference for TBDMS-cyanohydrin deprotection over the imidate salt formation.

The stereochemical assignments of (*S*)- and (*R*)-mchm^5^U (1 and 2, respectively) were confirmed by comparison of their CD spectra (ESI, Fig. S18†) with those reported earlier by Nawrot and Fu.^6,14^

## Conclusions

Two new, more reliable methods of preparation of the mixture of (*S*)-mchm^5^U (1) and (*R*)-mchm^5^U (2), based on the use of C5,1-functionalized uridines (the “malonate” 5 and formyl 9 derivatives) as the substrates, with subsequent chromatographic separation of the diastereomers, have been reported. These methods significantly improved the availability of these synthetically demanding nucleosides and will facilitate research on their presence and functions in native tRNAs and on the unique activities of the ALKB-like proteins involved in modulation of tRNA functions.

## Experimental

### General methods

Thin layer chromatography was done on silica gel coated plates (60F254, Merck), and Merck silica gel 60 (mesh 230–400, Merck) or Fluka silica gel 100 C_8_-RP were used for column chromatography. HPLC was performed with a Waters chromatograph equipped with a 996 spectral diode array detector and a preparative Ascentis® column (C18, 25 cm × 21.2 mm, 10 μm, SUPELCO). Separation was run at rt using water as an eluent. NMR spectra were recorded on 250 MHz or 700 MHz instruments (176 MHz for ^13^C NMR). Chemical shifts (*δ*) are reported in ppm relative to TMS (an internal standard) for ^1^H and ^13^C. The signal multiplicities are described as s (singlet), d (doublet), dd (doublet of doublets), t (triplet), q (quartet), m (multiplet), and bs (broad singlet). High-resolution mass spectrometry (HRMS) spectra were recorded using a Synapt G2Si mass spectrometer (Waters) equipped with an ESI source and a quadrupole-time-of-flight mass analyzer. The measurements were performed in a negative ion mode and the results were processed using the MassLynx 4.1 software (Waters). CD spectra were recorded on Spectrometer CD J-1000 JASCO in a 1 ml quartz cell. UV measurements were made using a Specord®50 PLUS spectrophotometer.

### 2′,3′,5′-tri-*O*-acetyl-*N*^3^-benzoyluridine-5-malonic acid diethyl ester (5)

To the solution of 2′,3′,5′-tri-*O*-acetyl-*N*^3^-benzoyl-5-bromouridine (4)^15^ (1.74 g, 3.15 mmol, 1 equiv.) in anhydrous THF (18 ml), diethyl malonate (535 μl, 3.5 mmol, 1.1 equiv.) and DBU (670 μl, 4.5 mmol, 1.4 equiv.) were added. The reaction mixture was stirred at room temperature for 15 h and then neutralized with AcOH. The solvent was evaporated under reduced pressure. The residue was dissolved in ethyl acetate (20 ml) and washed with water and brine. The organic layer was separated, dried over MgSO_4_ and concentrated *in vacuo*. The solid residue was applied on a silica gel column, further eluted with CHCl_3_ to afford compound 5 in 78% yield (1.55 g). TLC *R*_f_ = 0.29 (CH_2_Cl_2_/acetone 98 : 2, v/v). NMR (*δ* [ppm], CDCl_3_): ^1^H (700 MHz) 7.98 (s, 1H), 7.94–7.95 (m, 2H), 7.64–7.66 (m, 1H), 7.49–7.51 (m, 2H), 6.18 (d, 1H, *J* = 5.75 Hz), 5.39–5.43 (m, 2H), 4.87 (s, 1H), 4.35–4.38 (m, 3H), 4.17–4.26 (m, 4H), 2.16 (s, 3H), 2.12 (s, 3H), 2.04 (s, 3H), 1.28 (t, 3H, *J* = 7.00 Hz), 1.27 (t, 3H, *J* = 7.50 Hz). ^13^C (176 MHz) 170.26, 169.34, 169.23, 167.43, 166.92, 166.87, 160.74, 148.40, 139.13, 134.84, 130.80, 130.27, 128.80, 107.51, 87.07, 80.28, 72.60, 70.42, 62.97, 62.05, 46.76, 20.25, 20.15, 20.00, 13.58; HRMS calcd for C_29_H_31_N_2_O_14_ [M − H]^−^ 631.1775, found 631.1769 (ESI, Fig. S1 and S2†).

### 2′,3′,5′-tri-*O*-acetyl-*N*^3^-benzoyluridine-5-α-hydroxymalonic acid diethyl ester (6)

To a stirred solution of 2′,3′,5′-tri-*O*-acetyl-*N*^3^-benzoyluridine-5-malonic acid diethyl ester (5) (460 mg, 0.73 mmol, 1 equiv.) in 1,4-dioxane (6.4 ml), solid SeO_2_ (324 mg, 2.92 mmol, 4 equiv.) was added and the reaction mixture was refluxed for 18 h. The mixture was cooled down to room temperature and filtered. The filtrate was concentrated under reduced pressure, the residue was dissolved in ethyl acetate (10 ml) and washed with saturated NaHCO_3_, water and brine. The organic layer was dried over MgSO_4_ and concentrated. The product 6 was isolated by silica gel column chromatography (elution with 1–20% AcOEt in CH_2_Cl_2_) in 68% yield (322 mg). TLC *R*_f_ = 0.80 (AcOEt : CH_2_Cl_2_ 15 : 85, v/v). NMR (*δ* [ppm], DMSO-d_6_): ^1^H (250 MHz) 7.94–8.00 (m, 3H), 7.80–7.96 (m, 1H), 7.61–7.67 (m, 2H), 7.38 (s, 1H), 6.00 (d, 1H, *J* = 4.5 Hz), 5.53 (dd, 1H, *J* = 4.75 Hz, *J* = 6.25 Hz), 5.34–5.39 (m, 1H), 4.17–4.37 (m, 3H), 4.12 (q, 2H, *J* = 7.25 Hz), 2.06 (s, 3H), 2.05 (s, 3H), 2.02 (s, 3H), 1.13 (t, 3H, *J* = 7.25 Hz). ^13^C (176 MHz) 170.17, 169.54, 169.51, 169.39, 168.58, 168.36, 159.88, 148.43, 139.58, 139.92, 130.70, 130.38, 129.61, 113.38, 89.32, 79.55, 76.49, 72.68, 69.56, 62.64, 61.69, 20.49, 20.37, 20.34, 13.77; HRMS calcd for C_29_H_31_N_2_O_15_ [M − H]^−^ 647.1724, found 647.1724 (ESI, Fig. S3 and S4†).

### Cyclic intermediate – the lactone 7

To a solution of 6 (123 mg, 0.19 mmol, 1 equiv.) in anhydrous MeOH (2.8 ml), 2.2 M solution of MeONa in MeOH (370 μl) was added and the mixture was stirred at room temperature for 20 h. Then the mixture was diluted with MeOH (3 ml) and neutralized by DOWEX 50W (H^+^ form) resin. The resin was filtered off and the filtrate was concentrated. For spectral analyses small amount (*ca.* 15 mg) of the solid residue was applied on a column of silanized silica gel (100 C8-RP). The column was eluted with water to afford analytically pure 7. Because of the presence of diastereomers, some ^13^C NMR resonances were doubled (the secondary shifts in the ^13^C NMR spectrum are given in parentheses). TLC *R*_f_ = 0.82 (iPrOH/H_2_O, 7 : 2, v/v). NMR (*δ* [ppm], D_2_O): ^1^H (250 MHz) 7.96 (s, 0.5H), 7.95 (s, 0.5H), 5.92 (d, 0.5H, *J* = 1.5 Hz), 5.91 (d, 0.5H, *J* = 1.75 Hz), 4.27–4.31 (m, 1H), 4.09–4.20 (m, 2H), 3.87–3.92 (m, 1H), 3.79 (s, 3H), 3.72–3.77 (m, 1H). ^13^C (176 MHz) 171.76 (171.66), 170.89 (170.85), 163.33, 150.79 (150.77), 139.49 (139.46), 113.35 (133.23), 89.30 (89.24), 83.76 (83.74), 77.96 (77.94), 73.74 (73.65), 69.08 (69.04), 60.40 (60.39), 53.28 (53.27) (ESI, Fig. S5 and S6†).

### (*S*)- and (*R*)-5-carboxyhydroxymethyluridine (8)

The remaining amount of crude compound 7 (*vide supra*) was dissolved in 50% aqueous TFA (3.4 ml). The solution was stirred for 15 h at 60 °C and concentrated *in vacuo*. The residue was purified on a column of silanized silica gel (100 C8-RP) eluted with water. Compound 8 was obtained in 50% yield (30 mg, the yield refers to the starting compound 6). TLC *R*_f_ = 0.63 (iPrOH/H_2_O, 7 : 3, v/v, for both diastereoisomers); NMR (*δ* [ppm]): ^1^H (700 MHz, D_2_O) 8.04 (s, 0.5H), 8.03 (s, 0.5H), 5.85 (d, 0.5H, *J* = 4.2 Hz), 5.86 (d, 0.5H, *J* = 4.2 Hz), 5.03 (s, 0.5H), 5.04 (s, 0.5H), 4.28–4.30 (m, 1H), 4.17–4.19 (m, 1H), 4.07–4.08 (m, 1H), 3.88 (dd, 1H, *J* = 2.8 Hz, *J* = 12.6 Hz), 3.74–3.77 (m, 1H), ^1^H (700 MHz, DMSO-d_6_) 11.44 (s, 0.5H), 11.43 (s, 0.5H), 7.90 (s, 0.5H), 7.88 (s, 0.5H), 5.82 (s, 0.5H), 5.81 (s, 0.5H), 5.58 (bs, 1H), 5.38–5.41 (m, 1H), 5.11 (bs, 1H), 5.04 (bs, 1H), 4.75 (s, 0.5H), 4.74 (s, 0.5H), 4.02–4.06 (m, 1H), 3.94–3.98 (m, 1H), 3.85–3.88 (m, 1H), 3.53–3.65 (m, 2H). ^13^C (176 MHz, D_2_O) 174.50, 163.40 (163.35), 150.90, 140.69, 112.13, 89.26 (89.21), 83.70, 73.52, 68.70 (68.69), 66.45 (66.38), 60.01 (59.97); HRMS calcd for C_11_H_13_N_2_O_9_ [M − H]^−^ 317.0621, found 317.0623 (ESI, Fig. S7–S9†).

### Conversion of 8 into (*S*)-5-methoxycarbonylhydroxymethyluridine (1) and (*R*)-5-methoxycarbonylhydroxymethyluridine (2)

5-Carboxyhydroxymethyluridine (8) (27 mg, 0.085 mmol) was dissolved in 1 M HCl/MeOH (1.6 ml). The solution was stirred at room temperature for 2 h and concentrated *in vacuo*. The residue was co-evaporated three times with anhydrous toluene and, finally, with methanol. A sample was applied on a preparative C18 column (SUPELCO; Ascentis®, 25 cm/21.2 mm; 10 μm; flow 6 ml min^−1^) further eluted with water. After HPLC separation, 12 mg (43%) of (*S*)-mchm^5^U (1) and 14 mg (50%) of (*R*)-mchm^5^U (2) were obtained (*t*_R_ = 7.9 min and 9.4 min, respectively). TLC: *R*_f_ = 0.45 (*n*-BuOH/H_2_O, 85 : 15, v/v, for both diastereoisomers). Spectral data for 1: NMR (*δ* [ppm], D_2_O): ^1^H (700 MHz) 8.13 (s, 1H), 5.96 (d, 1H, *J* = 4.2 Hz), 5.15 (s, 1H), 4.39 (dd, 1H, *J* = 4.2 Hz, *J* = 5.6 Hz), 4.28 (t, 1H, *J* = 5.6 Hz), 4.18–4.20 (m, 1H), 3.90 (dd, 1H, *J* = 2.8 Hz, *J* = 12.6 Hz), 3.86 (dd, 1H, *J* = 4.2 Hz, *J* = 12.6 Hz), 3.82 (s, 3H). ^13^C NMR (176 MHz) 173.69, 163.81, 151.36, 141.14, 112.45, 89.64, 84.09, 73.89, 69.09, 67.08, 60.40, 53.14; HRMS calcd for C_12_H_15_N_2_O_9_ [M − H]^+^ 331.0778, found 331.0783. Spectral data for 2: NMR (*δ* [ppm], D_2_O): ^1^H (700 MHz) 8.14 (s, 1H), 5.96 (d, 1H, *J* = 4.2 Hz), 5.16 (s, 1H), 4.40 (dd, 1H, *J* = 4.2 Hz, *J* = 5.6 Hz), 4.29 (t, 1H, *J* = 5.6 Hz), 4.18–4.19 (m, 1H), 3.99 (dd, 1H, *J* = 2.8 Hz, *J* = 12.6 Hz), 3.86 (dd, 1H, *J* = 3.5 Hz, *J* = 12.6 Hz), 3.82 (s, 3H). ^13^C (176 MHz) 173.68, 163.82, 151.39, 141.23, 112.34, 89.69, 84.02, 73.78, 69.02, 67.05, 60.31, 53.14; HRMS calcd for C_12_H_15_N_2_O_9_ [M − H]^−^ 331.0778, found 331.0784; UV (H_2_O) *λ*_max_ = 266 (*ε*_266_ = 9553 l mol^−1^ cm^−1^, *ε*_260_ = 8592 l mol^−1^ cm^−1^) (ESI, Fig. S10–S13†).

### (*S*)- and (*R*)-2′,3′-*O*-isopropylidene-5′-*O-tert*-butyldimethylsilyl-5-(*O-tert*-butyldimethylsilyl)cyanohydroxymethyluridine (10b)

To a solution of 2′,3′-*O*-isopropylidene-5-formyluridine (9)^18^ (1.55 g, 4.98 mmol, 1 equiv.) in anhydrous acetonitrile (62 ml), TBDMS-CN (2.09 g, 14.94 mmol, 3 equiv) and triethylamine (2.06 ml, 14.94 mmol, 3 equiv.) were added. The mixture was stirred at room temperature for 2 h and concentrated under reduced pressure. The crude product was purified by silica gel column chromatography using a 0–5% gradient of methanol in chloroform as an eluent. Compound 10b was obtained in 80% yield (2.26 g). Because of the presence of diastereomers some ^13^C NMR resonances were doubled (the secondary shifts in the ^13^C NMR spectrum are given in parentheses). *R*_f_ = 0.29 (CH_2_Cl_2_/acetone 98 : 2, v/v), 0.53 (hexane/ethyl acetate 3 : 1 v/v). NMR (*δ* [ppm], CDCl_3_): ^1^H (700 MHz) 0.06 (s, 1.5H), 0.08 (s, 1.5H), 0.08 (s, 3H), 0.18 (s, 1.5H), 0.19 (s, 1.5H), 0.25 (s, 1.5H), 0.27 (s, 1.5H), 0.86 (s, 4.5H), 0.88 (s, 4.5H), 0.93 (s, 9H), 1.36 (s, 1.5H), 1.37 (s, 1.5H), 1.58 (s, 3H), 4.33–4.35 (m, 0.5H), 4.44–4.45 (m, 0.5H), 4.72–4.73 (m, 0.5H), 4.88–4.90 (m, 1H), 5.48 (s, 0.5H), 5.50 (s, 0.5H), 5.69 (d, 0.5H, *J* = 2.37 Hz), 5.71 (d, 0.5H, *J* = 2.46 Hz), 7.74 (s, 0.5H), 7.82 (s, 0.5H), 8.90 (s, 0.4H), 8.86 (s; 0.4H); ^13^C (176 MHz) −4.60 (−4.49), −4.35 (−4.23), 19.11 (19.16), 19.28 (19.36), 26.15 (25.24), 26.51 (26.56), 26.91 (26.85), 29.08 (29.16), 58.39 (58.47), 64.76 (64.66), 82.51 (82.26), 85.99 (86.70), 88.79 (89.06), 96.53 (96.09), 110.82 (111.55), 114.76 (115.12), 118.61 (118.66), 140.64 (141.43), 150.38 (150.43), 161.87 (161.90). HRMS calcd for C_26_H_45_N_3_O_7_Si [M − H]^−^ 567.2796, found 567. 2722 (ESI, Fig. S15 and S16†).

### Conversion of 10b into (*S*)-5-methoxycarbonylhydroxymethyl-uridine (1) and (*R*)-5-methoxycarbonylhydroxymethyluridine (2)

Compound 10b (80 mg, 0.14 mmol) was dissolved in cold (an ice bath) 4 M HCl/MeOH (8 ml, prepared by dropwise addition of AcCl to cold anhydrous MeOH). The reaction vessel was moved to a refrigerator (set at 5 °C) and the mixture was stirred for 2 h (TLC control, isopropanol/water 7 : 3, v/v, *R*_f_ = 0.88). The volatile components were removed *in vacuo* and the residue was three times co-evaporated with methanol and dried under reduced pressure to remove HCl. The resultant imidate salt 11 was dissolved in ice water (8 ml) and stored at 5 °C (refrigerator conditions) for 2 h (TLC control; *n*-BuOH/H_2_O, 85 : 15, v/v, *R*_f_ = 0.45 for both diastereomers 1 and 2). The sample was lyophilized and applied on a preparative C18 column (SUPELCO; Ascentis® (25 cm/21.2 mm; 10 μm; flow 6 ml min^−1^) further eluted with water. The isomers 1 and 2 were isolated in amounts 11 mg and 12 mg, respectively (24% and 26%, calculated over starting 10, *t*_R_ = 7.9 min and 9.4 min). The spectral data conformed to those recorded for 1 and 2 obtained from 8.

## Conflicts of interest

There are no conflicts to declare.

## Supplementary Material

RA-009-C9RA08548C-s001

## References

[cit1] Boccaletto P., Machnicka M. A., Purta E., Piatkowski P., Baginski B., Wirecki T. K., de Crecy-Lagard V., Ross R., Limbach P. A., Kotter A., Helm M., Bujnicki J. M. (2017). Nucleic Acids Res..

[cit2] Agris P. F., Eruysal E. R., Narendran A., Vare V. Y. P., Vangaveti S., Ranganathan S. V. (2018). RNA Biol..

[cit3] Duechler M., Leszczynska G., Sochacka E., Nawrot B. (2016). Cell. Mol. Life Sci..

[cit4] Torres A. G., Batlle E., de Paoplana L. R. (2014). Trends Mol. Med..

[cit5] van den Born E., Vagbo C. B., Songe-Moller L., Leihne V., Lien G. F., Leszczynska G., Malkiewicz A., Krokan H. E., Kirpekar F., Klungland A., Falnes P. (2011). Nat. Commun..

[cit6] Fu Y., Dai Q., Zhang W., Ren J., Pan T., He Ch. (2010). Angew Chem. Int. Ed. Engl..

[cit7] Kawakami M., Nishio K., Takemura S., Kondo T., Goto T. (1979). Nucleic Acids Symp. Ser..

[cit8] Kawakami M., Takemura S., Kondo T., Fukami T., Goto T. (1988). J. Biochem..

[cit9] Leihne V., Kirpekar F., Vagbo C. B., van der Born E., Krokan H. E., Grini P. E., Meza T. J., Falnes P. O. (2011). Nucleic Acids Res..

[cit10] Fedeles B. I., Singh V., Delaney J. C., Li D., Essigmann J. M. (2015). J. Biol. Chem..

[cit11] Fu D., Brophy J. A. N., Chan C. T. Y., Atmore K. A., Begley U., Paules R. S., Dedon P. C., Begley T. J., Samson L. D. (2010). Mol. Cell. Biol..

[cit12] Shimada K., Nakamura M., Anai S., De Velasco M., Tanaka M., Tsujikawa K., Ouji Y., Konishi N. (2009). Cancer Res..

[cit13] Monies D., Vagbo C. B., Al-Owain M., Alhomaidi S., Alkuraya F. S. (2019). Am. J. Hum. Genet..

[cit14] Nawrot B., Malkiewicz A. (1989). Nucleosides Nucleotides.

[cit15] Inoue H., Saito N., Ueda T. (1986). Chem. Pharm. Bull..

[cit16] Patel R. M., Puranik V. G., Argade N. P. (2011). Org. Biomol. Chem..

[cit17] Schroder A. S., Steinbacher J., Steigenberger B., Gnerlich F. A., Schiesser S., Pfaffeneder T., Carell T. (2014). Angew. Chem. Int. Ed..

[cit18] Leszczynska G., Pięta J., Wozniak K., Malkiewicz A. (2014). Org. Biomol. Chem..

[cit19] Anderson K. , Chen Y., Chen Z., Luk K.-Ch., Rossman P. L., Sun H. and Wovkulich P. M., *US Pat.* 2012/184542 A1, 2012

[cit20] Walker J. R., Alshafie N., Nieves N., Clagett-Damw J. A., Abou-Issab H., Curley R. W. (2006). Bioorg. Med. Chem..

[cit21] Herranz R., Castro-Pichel J., Vinuesa S., Garcia-Lopez M. T. (1990). J. Org. Chem..

[cit22] Ziora Z., Kasai S., Hidaka K., Nagamine A., Kimura T., Hayashi Y., Kiso Y. (2007). Bioorg. Med. Chem. Lett.

