# Supplementary material for: Novel entry to the synthesis of (S)- and (R)-5-methoxycarbonylhydroxymethyluridines – a diastereomeric pair of wobble tRNA nucleosides
Source: RSC Adv. 2019 Dec 6;9(69):40507–12. doi: 10.1039/c9ra08548c (PMC9076229; doi:10.1039/c9ra08548c)
Supplement: RA-009-C9RA08548C-s001 [file RA-009-C9RA08548C-s001.pdf]

Electronic Supplementary Material (ESI) for RSC Advances.
This journal is © The Royal Society of Chemistry 2019

Novel entry to the synthesis of (*S*)- and (*R*)-5-methoxycarbonylhydroxymethyluridines – diastereomeric pair of wobble tRNA nucleosides

Robert Borowski, Agnieszka Dziergowska, Grazyna Leszczynska, Elzbieta Sochacka

Institute of Organic Chemistry, Lodz University of Technology, Zeromskiego 116, 90-924 Lodz, Poland

Table of contents:

1. ^{1}H and ^{13}C spectra of compounds **1**, **2**, **5-8**, and **10a-b** S2-S17
2. RP-HPLC chromatograph of (*S*)-mchm^{5}U and (*R*)-mchm^{5}U S18
3. CD spectrum of (*S*)-mchm^{5}U and (*R*)-mchm^{5}U S19

Fig. S1. ^1H NMR (700 MHz, $CDCl_3$) spectrum of 2′,3′,5′-tri-*O*-acetyl-*N*[3]-benzoyluridine-5-malonic acid diethyl ester (**5**).

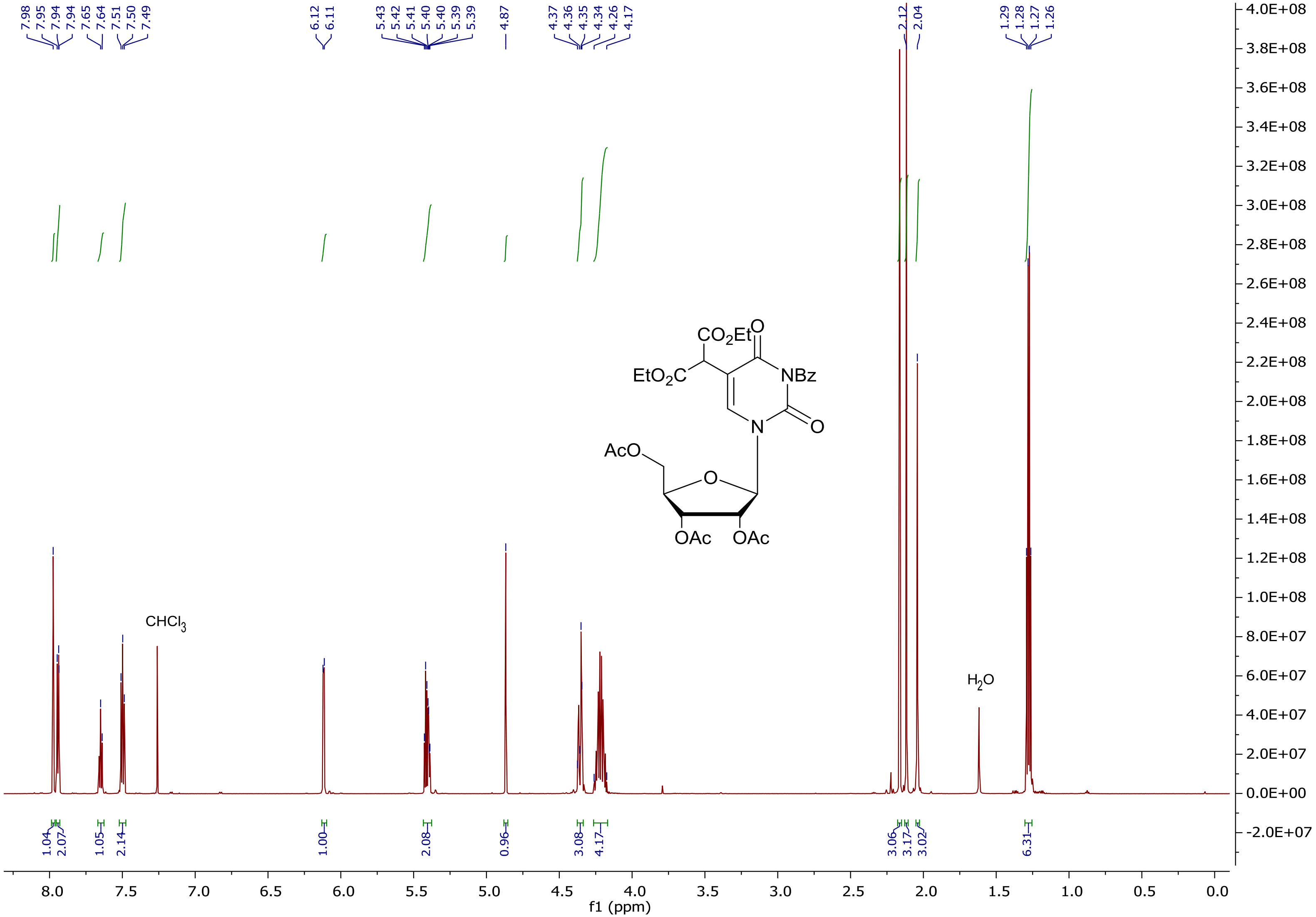

Fig. S2. ^{13}C NMR (176 MHz, $CDCl_3$) spectrum of 2′,3′,5′-tri-*O*-acetyl-*N*[3]-benzoyluridine-5-malonic acid diethyl ester (**5**).

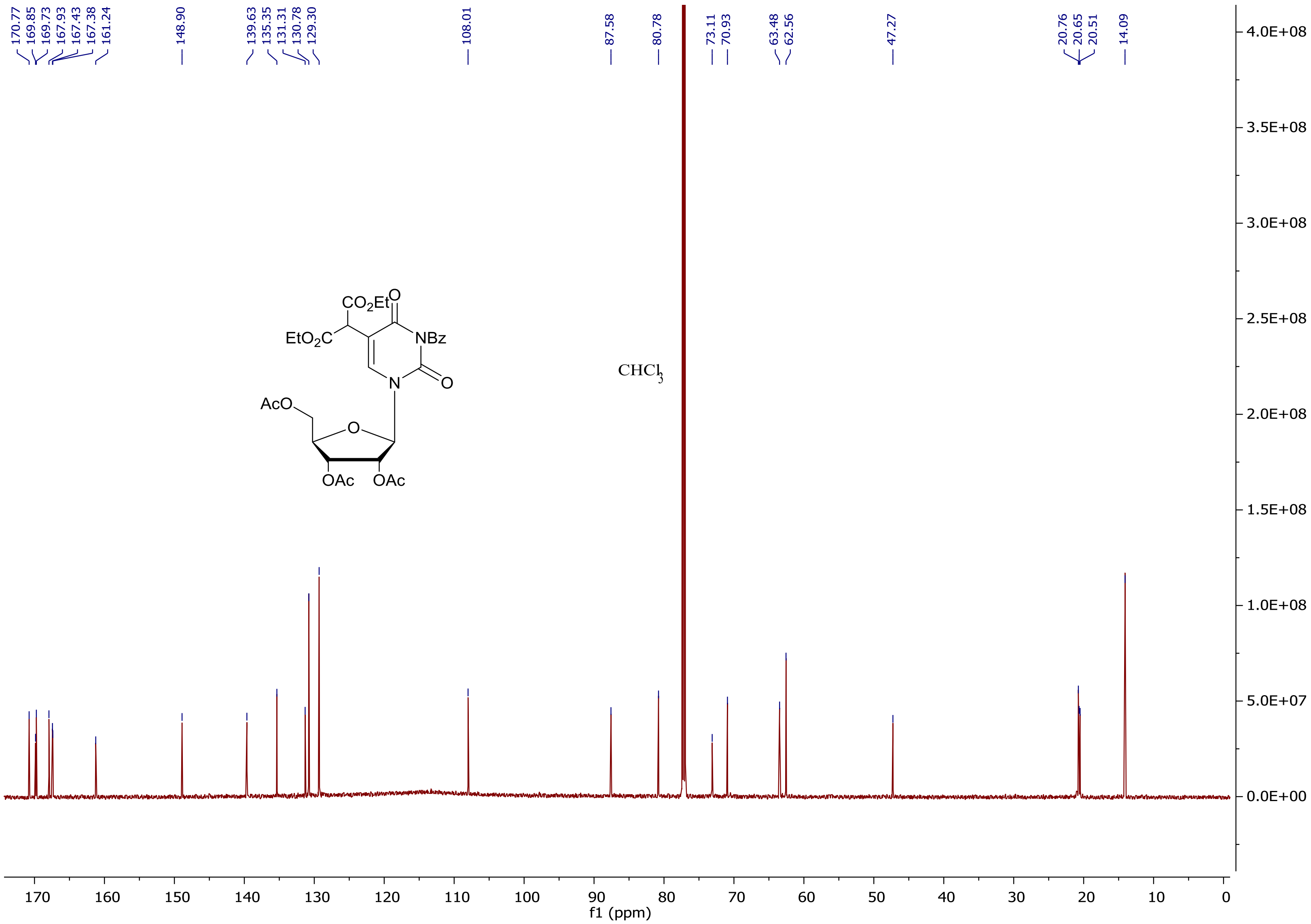

Fig.S3. ^{1}H NMR (250 MHz, DMSO – d6) spectrum of 2′,3′,5′-tri-*O*-acetyl-*N*^{3}-benzoyluridine-5-α-hydroxymalonic acid diethyl ester (**6**)

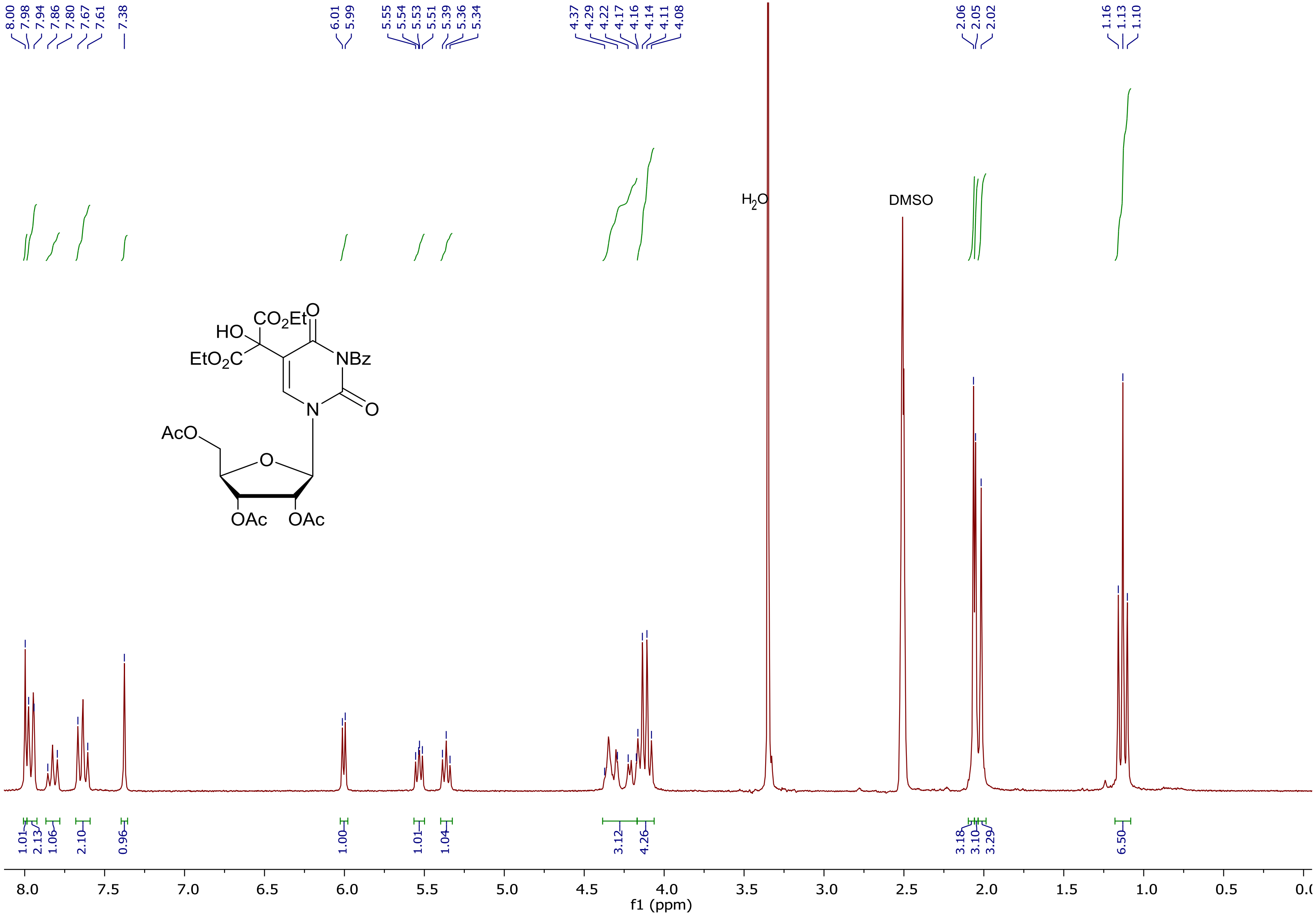

Fig.S4. ^{13}C NMR (176 MHz, DMSO – d6) spectrum of 2′,3′,5′-tri-*O*-acetyl-*N*^{3}-benzoyluridine-5-α-hydroxymalonic acid diethyl ester (**6**)

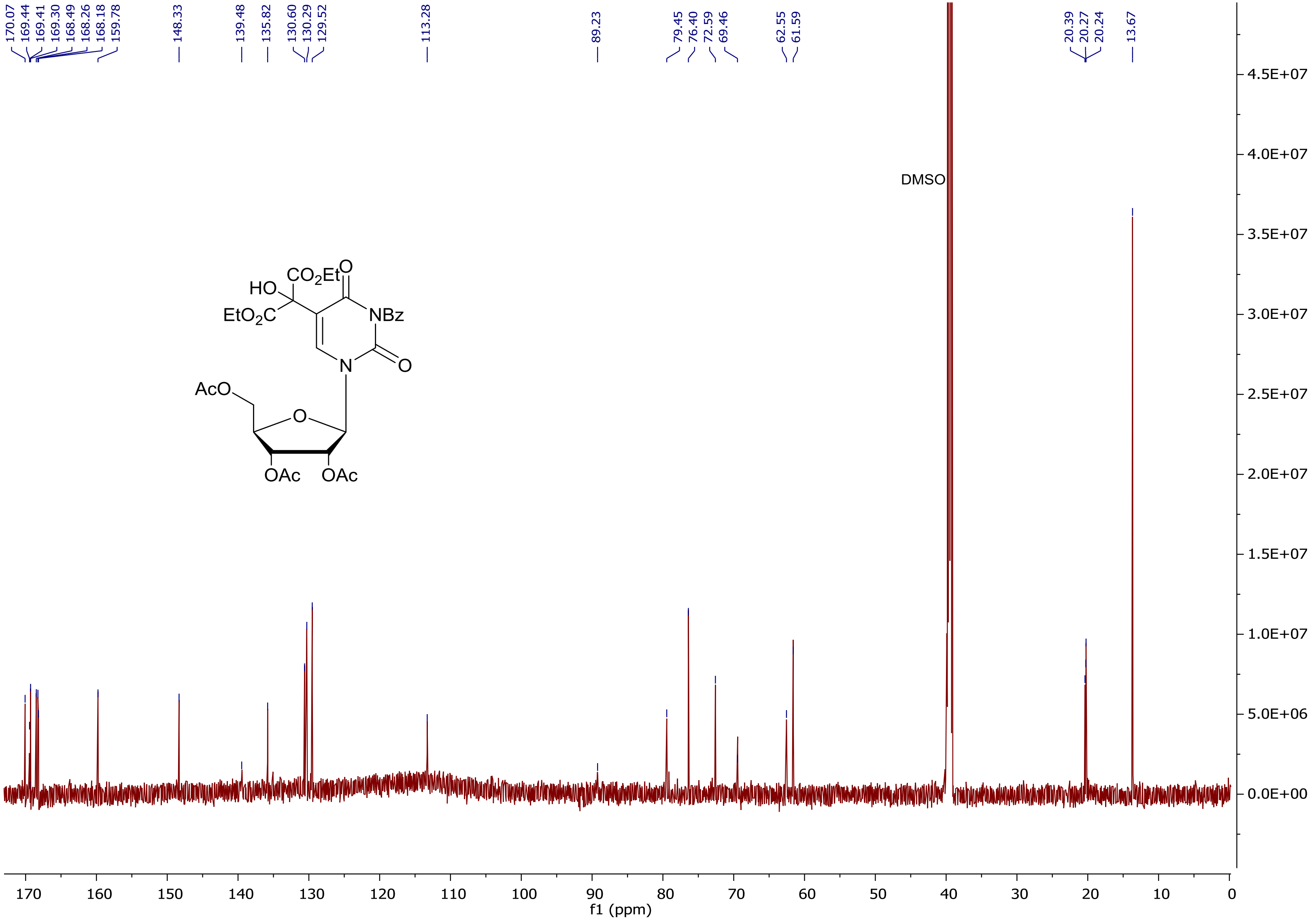

Fig.S5. ^{1}H NMR (250 MHz, D_2O) spectrum of lactone-containing intermediate **7**

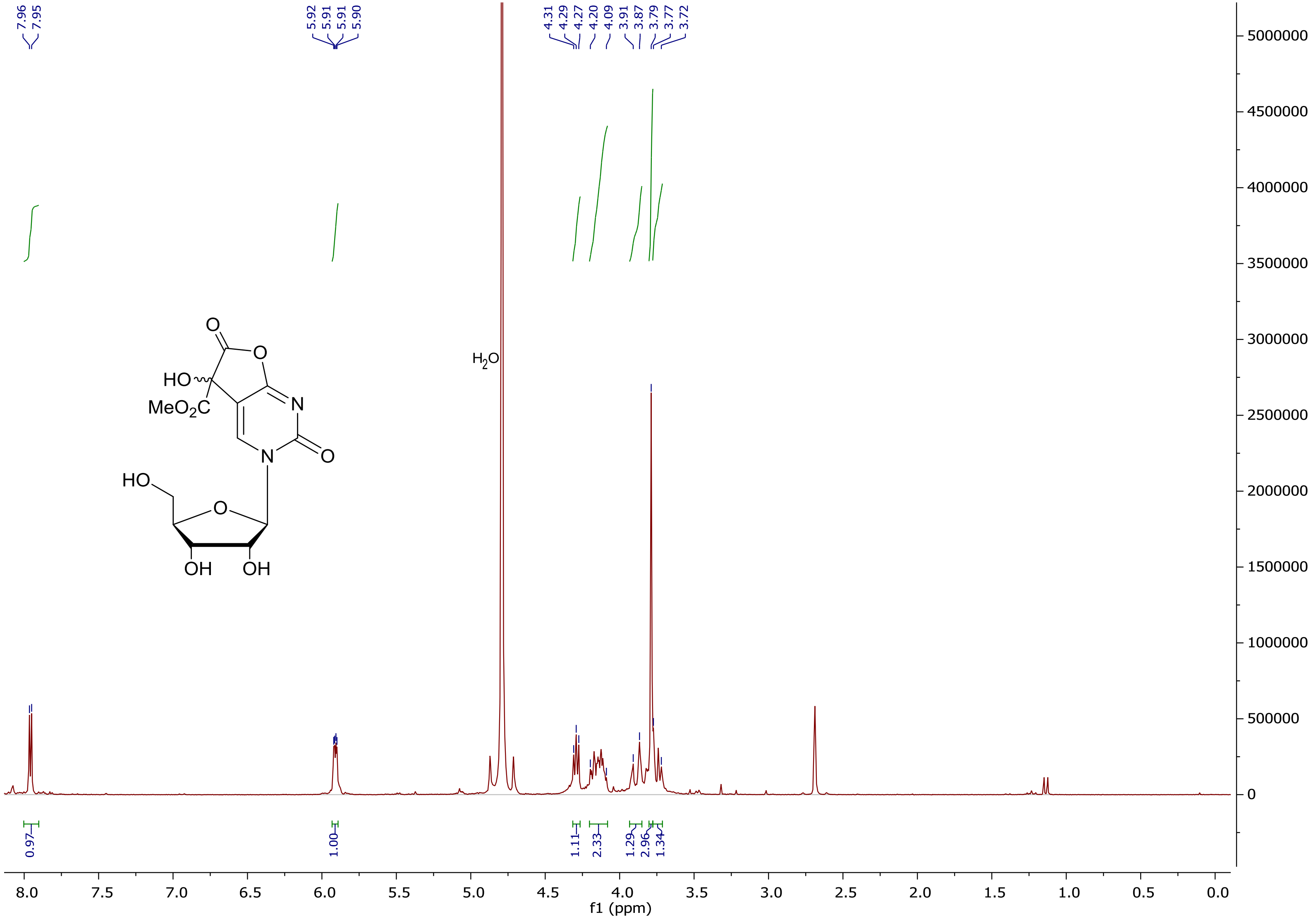

Fig.S6. ^{13}C NMR (176 MHz, D_2O) spectrum of lactone-containing intermediate **7**

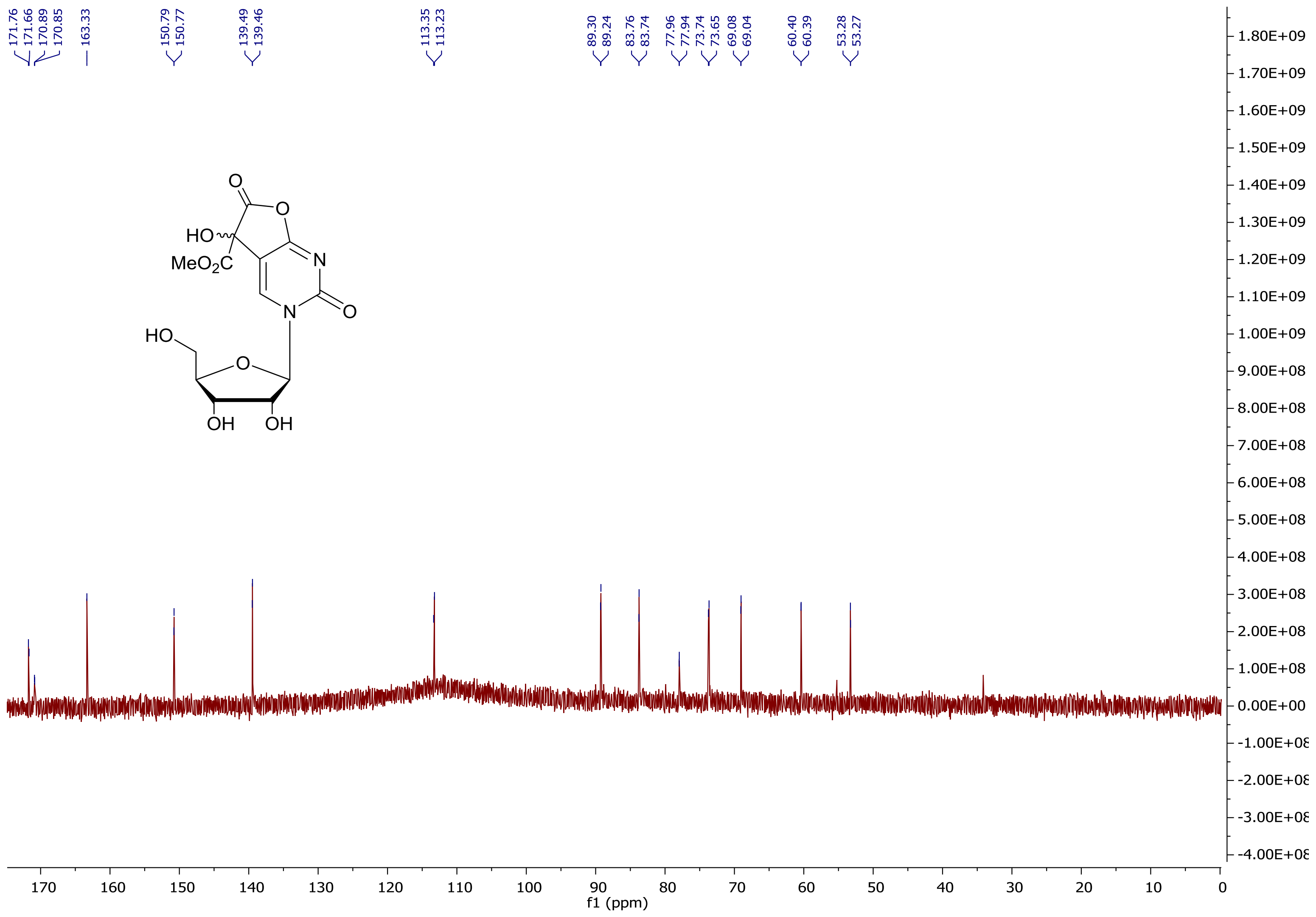

Fig.S7 ^{1}H NMR (700 MHz, D_2O) spectrum of (*S*)- and (*R*)-5-carboxyhydroxymethyluridine (**8**).

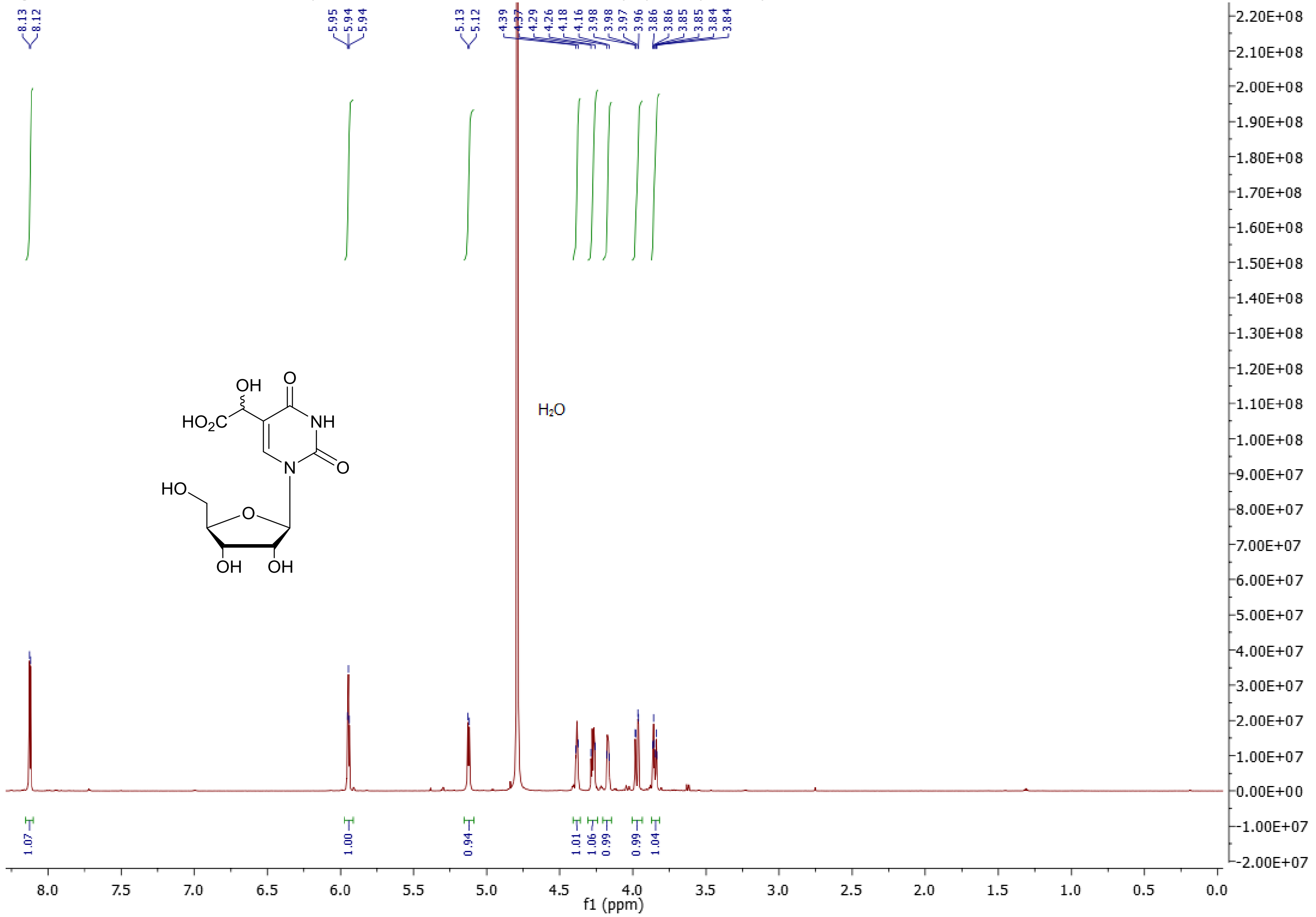

Fig.S8 ^{13}C NMR (176 MHz, D_2O) spectrum of (*S*)- and (*R*)-5-carboxyhydroxymethyluridine (**8**).

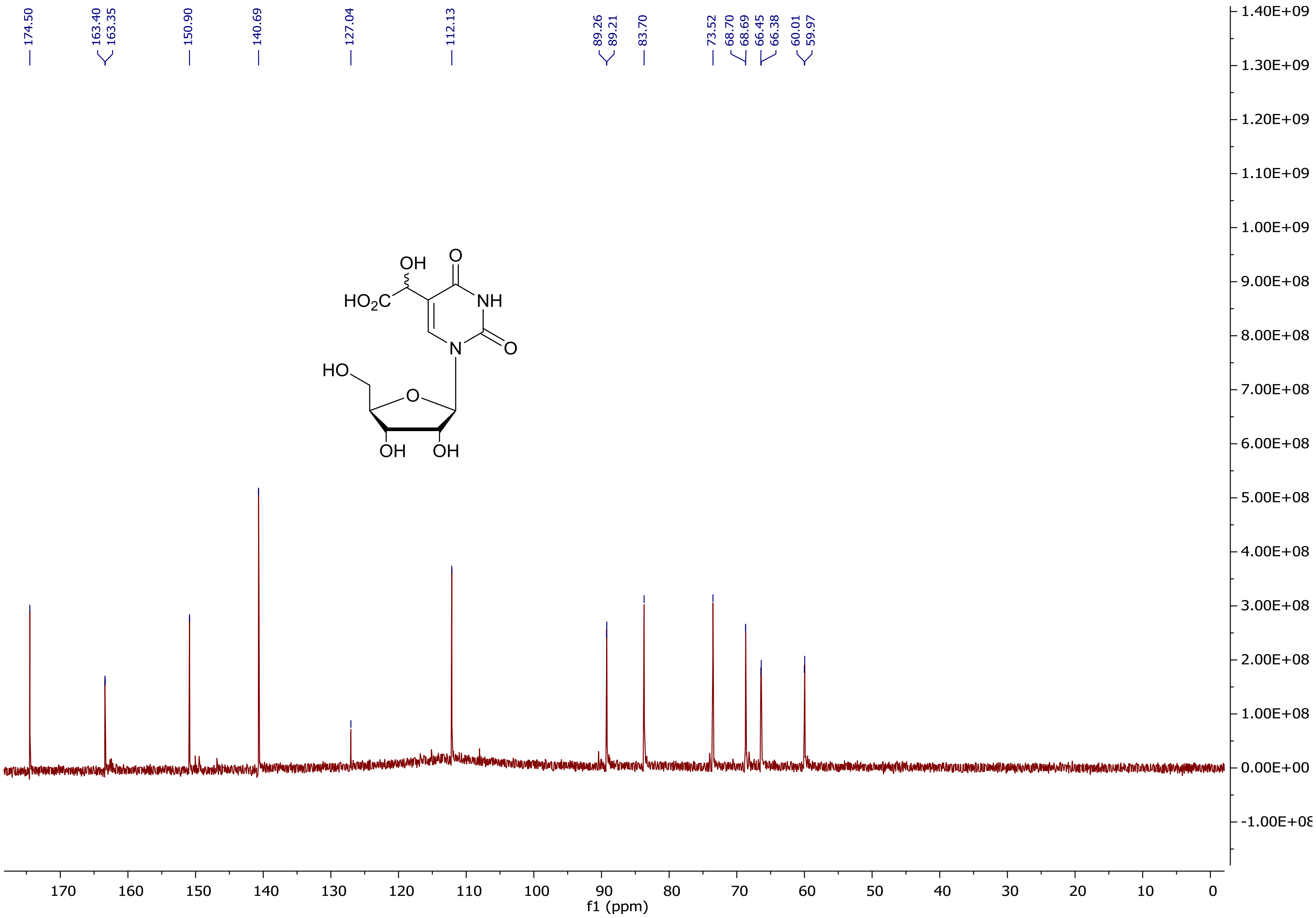

Fig.S9. ^{1}H NMR (700 MHz, DMSO – d6) spectrum of (*S*)- and (*R*)-5-carboxyhydroxymethyluridine (**8**).

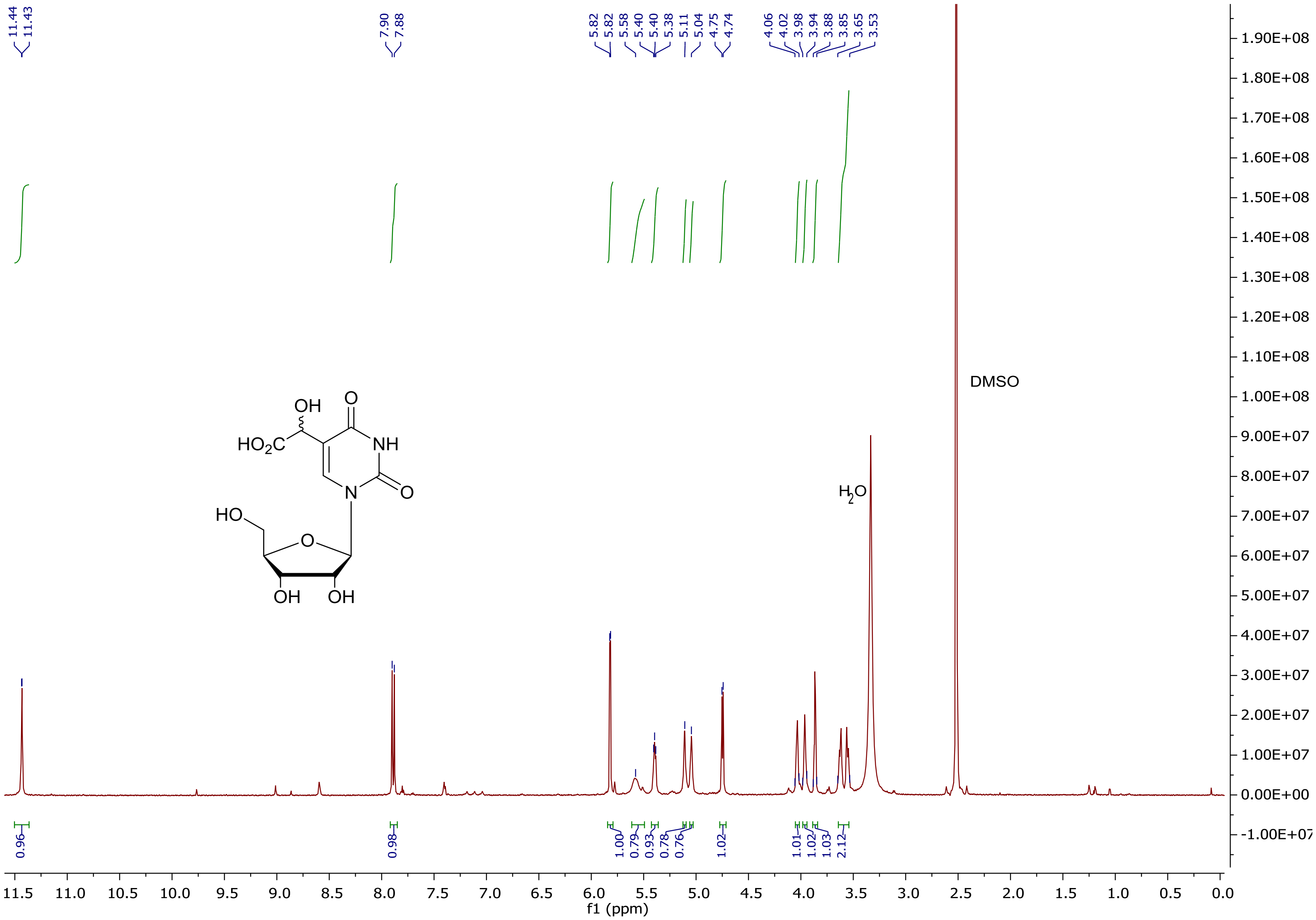

Fig.S10. ^{1}H NMR (700 MHz, D_2O) spectrum of (*S*)-5-methoxycarbonylhydroxymethylouridine (**1**).

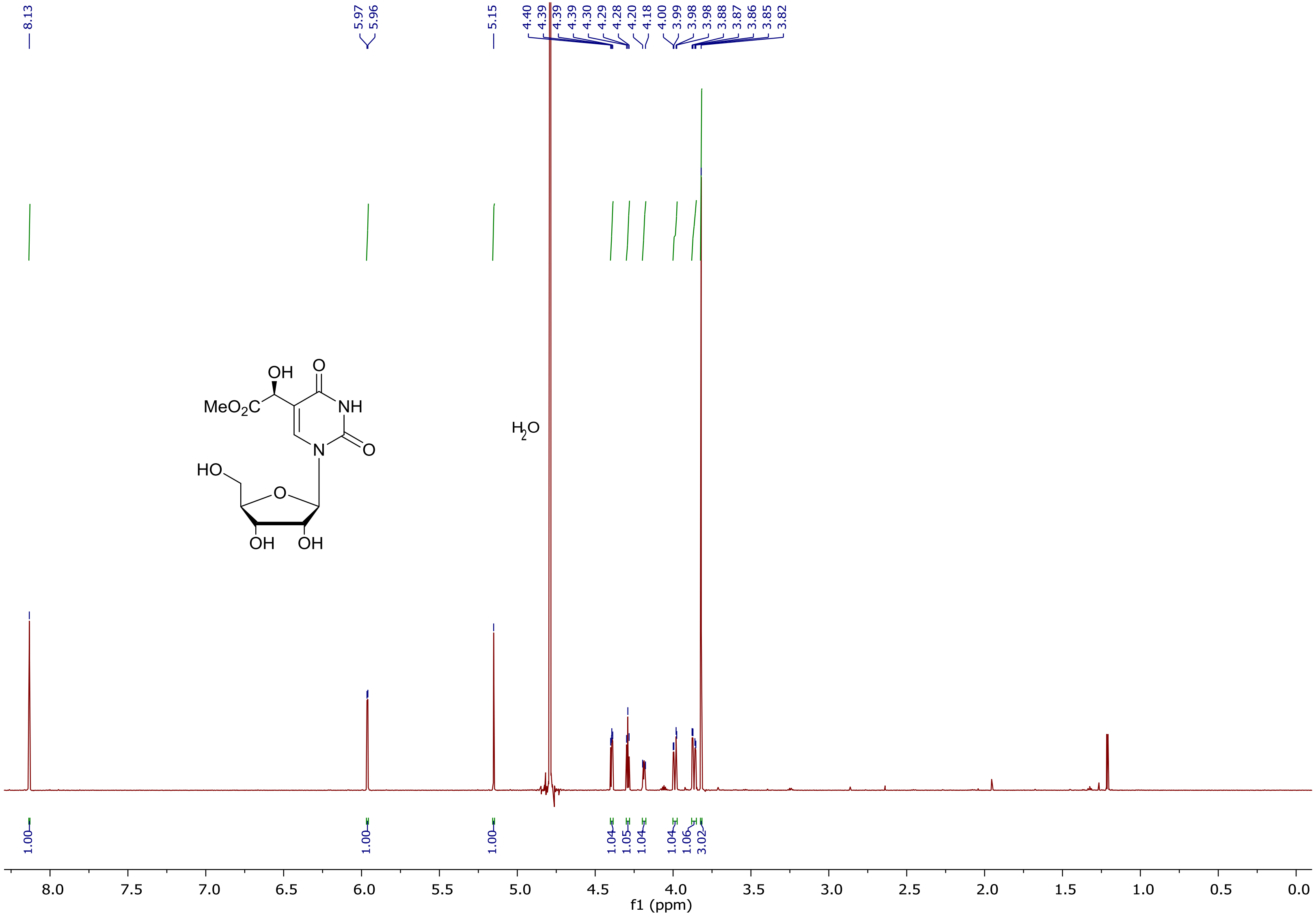

Fig.S11. ^{13}C NMR (176 MHz, D_2O) spectrum of (*S*)-5-methoxycarbonylhydroxymethylouridine (**1**).

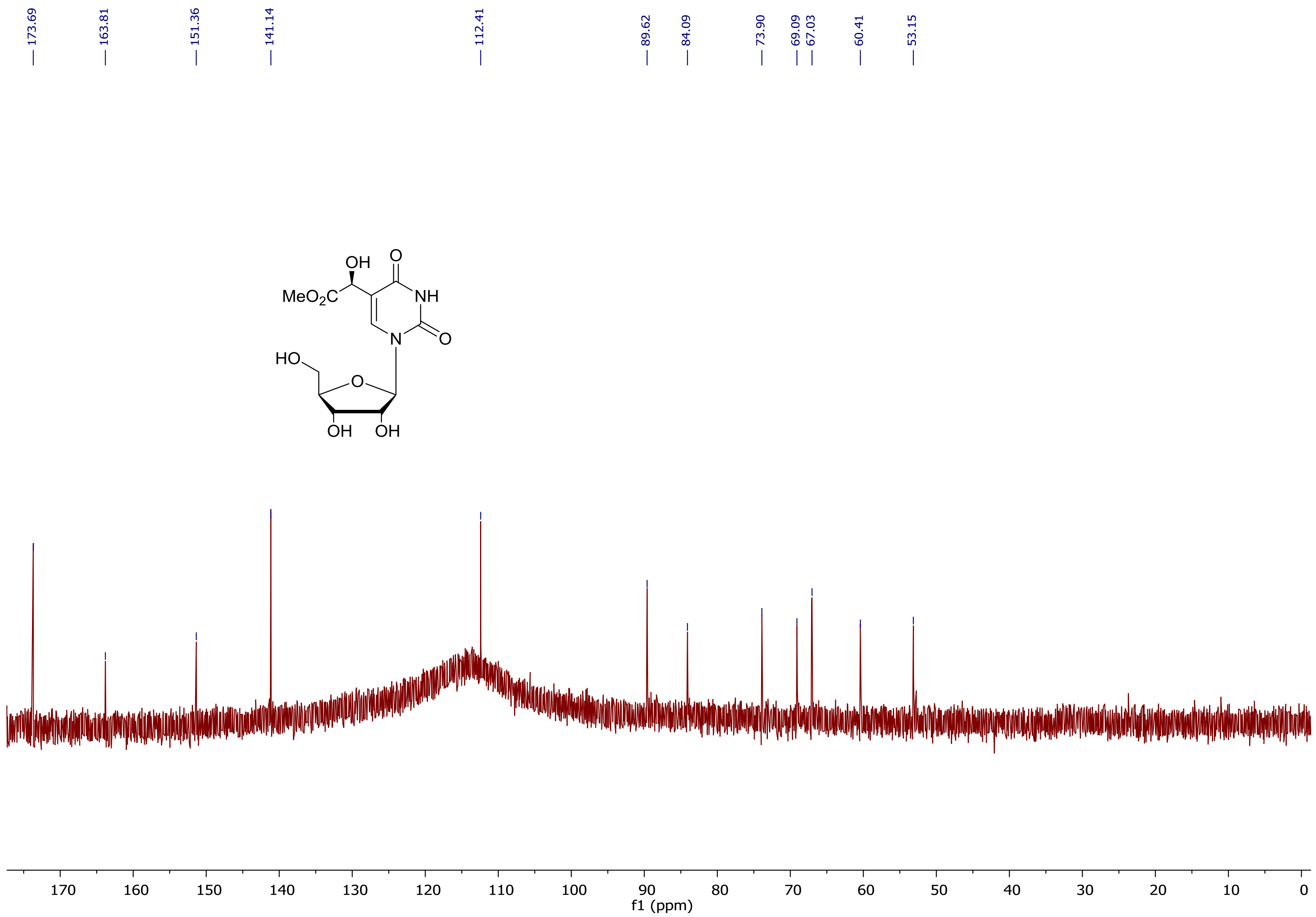

Fig.S12. ^{1}H NMR (700 MHz, D_2O) spectrum of (*R*)-5-methoxycarbonylhydroxymethylouridine (**2**).

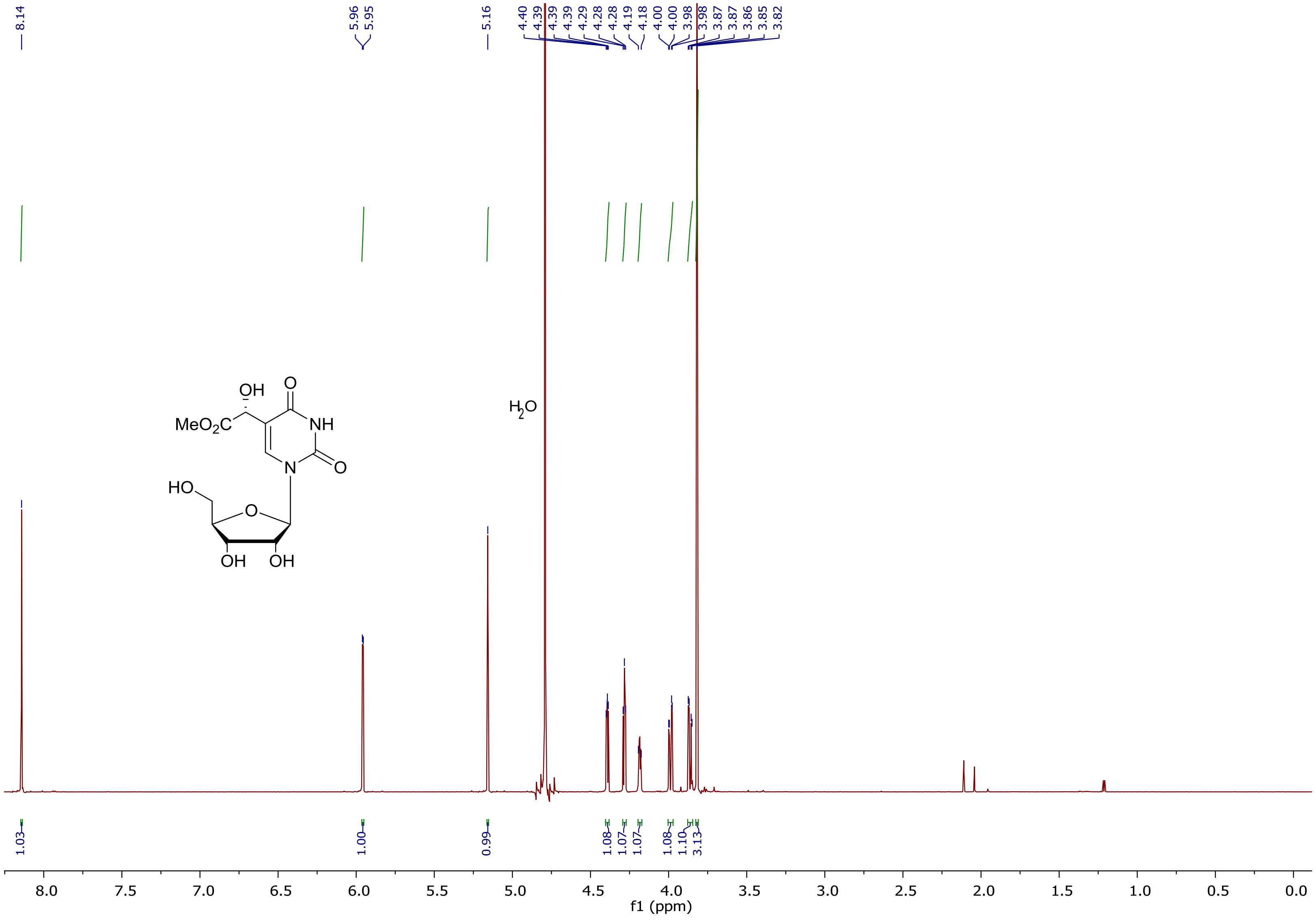

Fig.S13. ^{13}C NMR (176 MHz, D_2O) spectrum of (*R*)-5-methoxycarbonylhydroxymethylouridine (**2**).

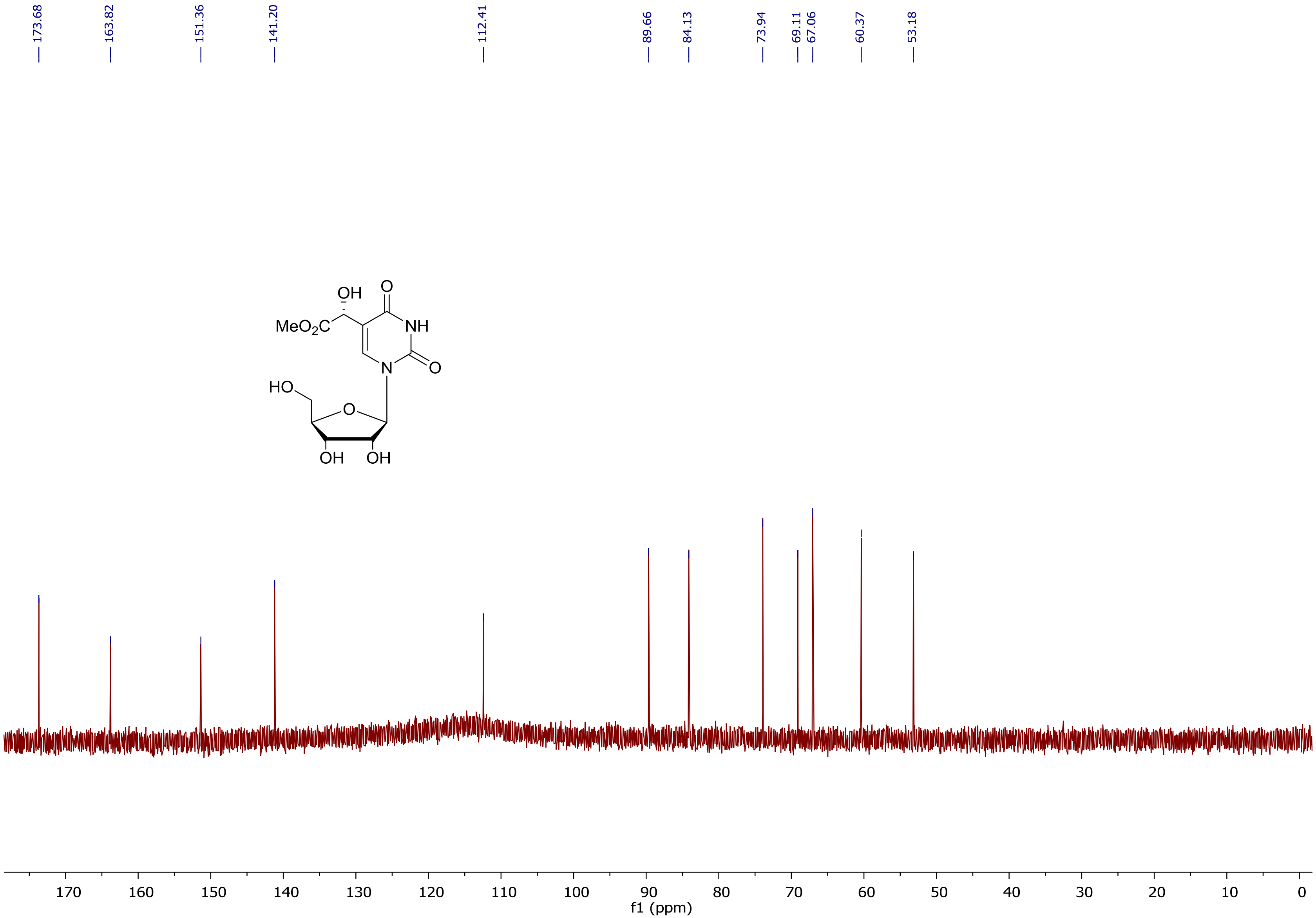

Fig.S14. ^{1}H NMR (700 MHz, CDCl3) spectrum of (*R*)- and (*S*)-*O*-(*tert*-butyldimethylsilyl)cyanohydrin of 2’,3’-*O*-isopropylidene-5-formyluridine (**10 a**).

NMR (δ [ppm], CDCl3): ^{1}H (700 MHz) 0,18 (s, 1.5H), 0.19 (s, 1.5H), 0.25 (s, 1.5H), 0,26 (s, 1.5H), 0.92 (s, 4.5H), 0.93 (s, 4.5H), 1.37 (s, 3H), 1.59 (s, 1.5H), 1.60 (s, 1.5H),2.58 (bs,0.4H) 2.63 (bs, 0.4H),3.82-3.84 (m, 1H), 3.94- 3.97 (m, 1H), 4.33- 4.34 (m, 0.5H), 4.41- 4.41 (m, 0.5H), 4.93-4.96 (dd, 0.5H, *J*=2.77 Hz, *J*=6.27 Hz), 4.96-4.98 (m, 1.4H), 5.49 (d, 0.5H, *J*=0.81 Hz), 5.50 (d, 0.5H, *J*=0,88 Hz),5.72 (d, 0.5H, *J* = 2.44 Hz), 5.76 (d, 0.5H, *J* = 2.82 Hz), 7.90 (s, 0.5H), 7.94 (s, 0.5H), 9,23 (bs, 1H).

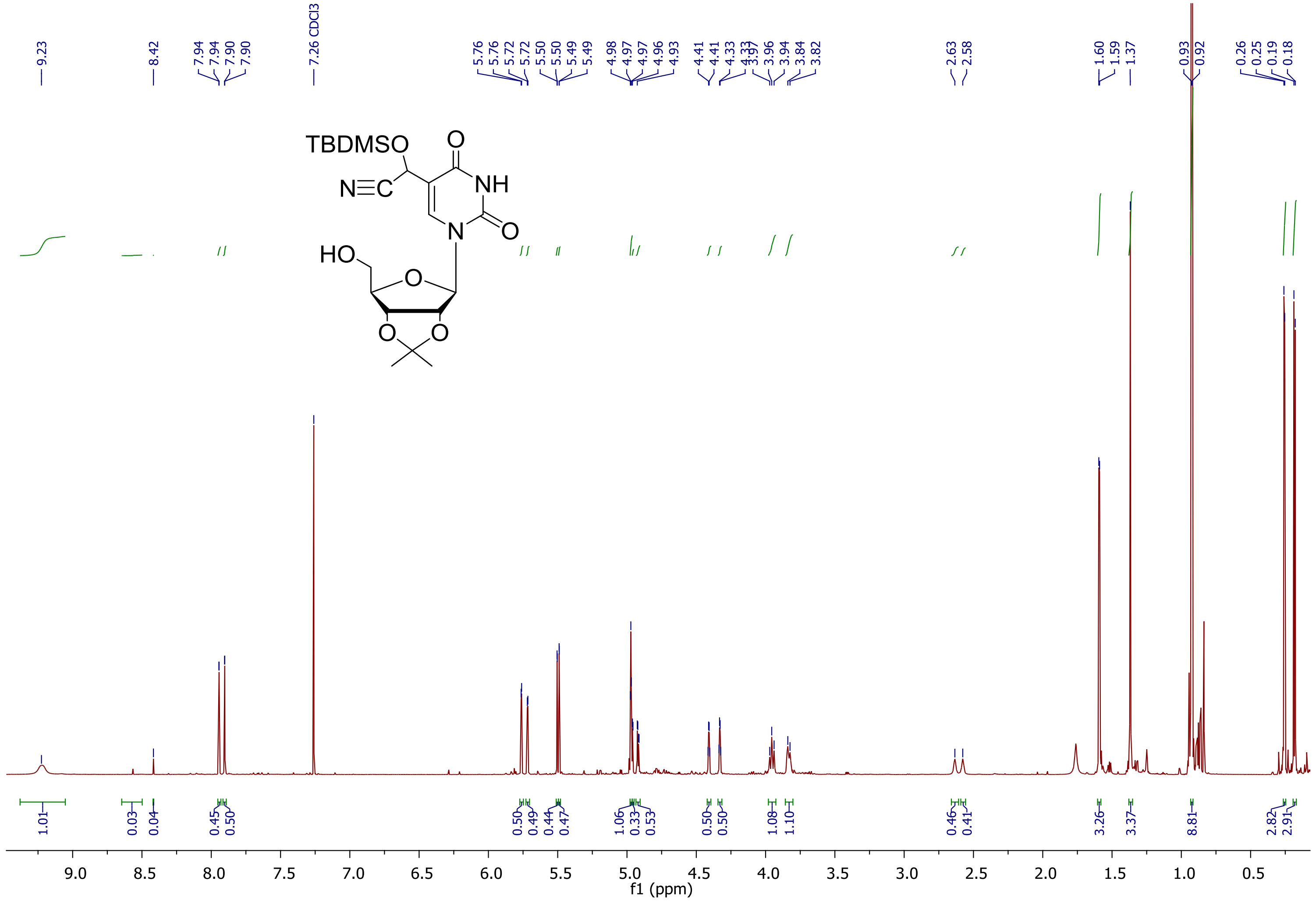

Fig.S15. ^{1}H NMR (700 MHz, $CDCl_3$) spectrum of (*S*)- and (*R*)-2',3'-*O*-izopropylidene-5′-*O*-*tert*-butyldimethylsilyl-5-(*O*-*tert*-butyldimethylsilyl)cyanohydroxymethyluridine (**10b**).

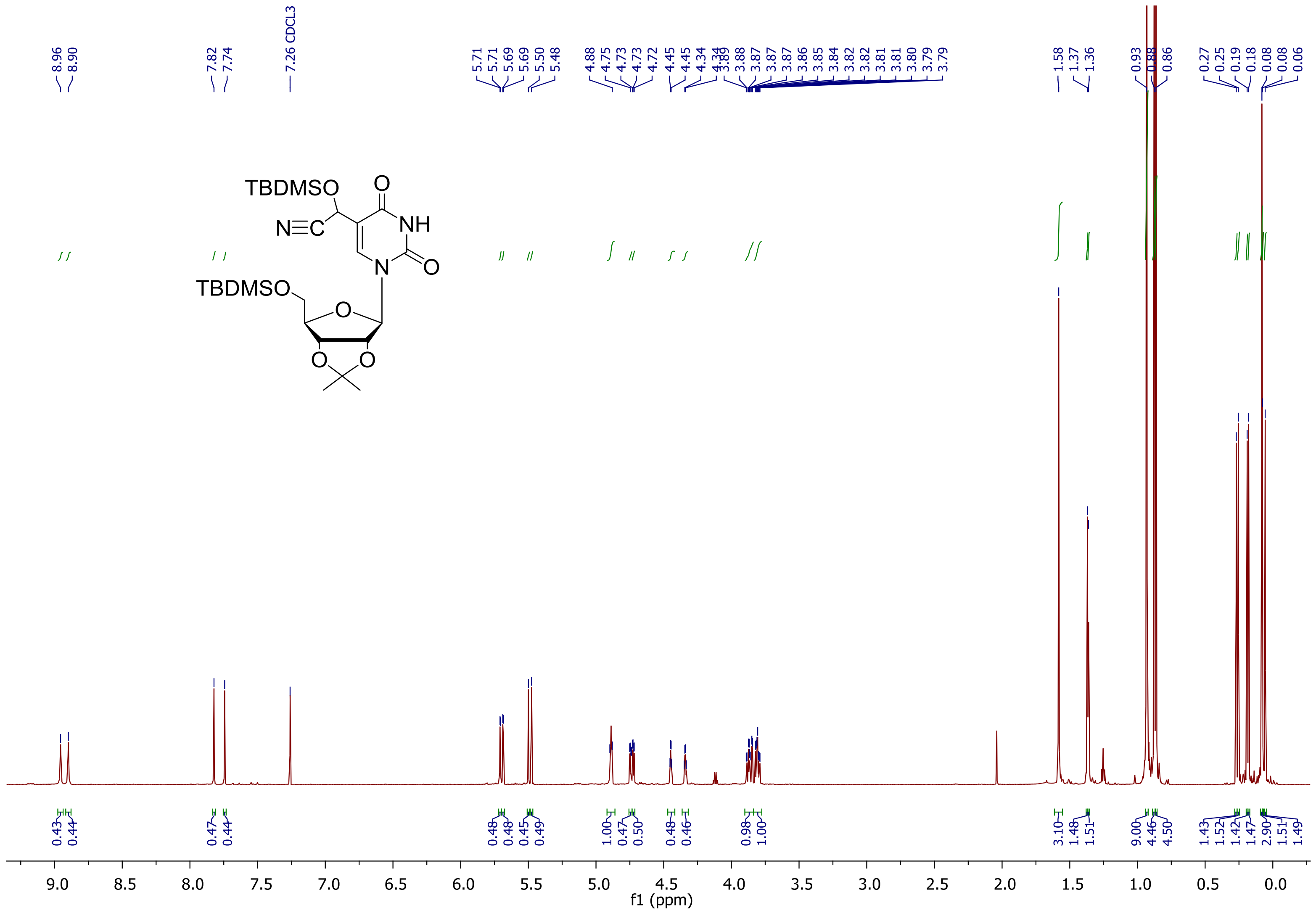

Fig.S16. ^{13}C NMR (176 MHz, $CDCl_3$) spectrum of (*S*)- and (*R*)-2',3'-*O*-izopropylidene-5'-*O*-*tert*-butyldimethylsilyl-5-(*O*-*tert*-butyldimethylsilyl)cyanohydroxymethyluridine (**10b**).

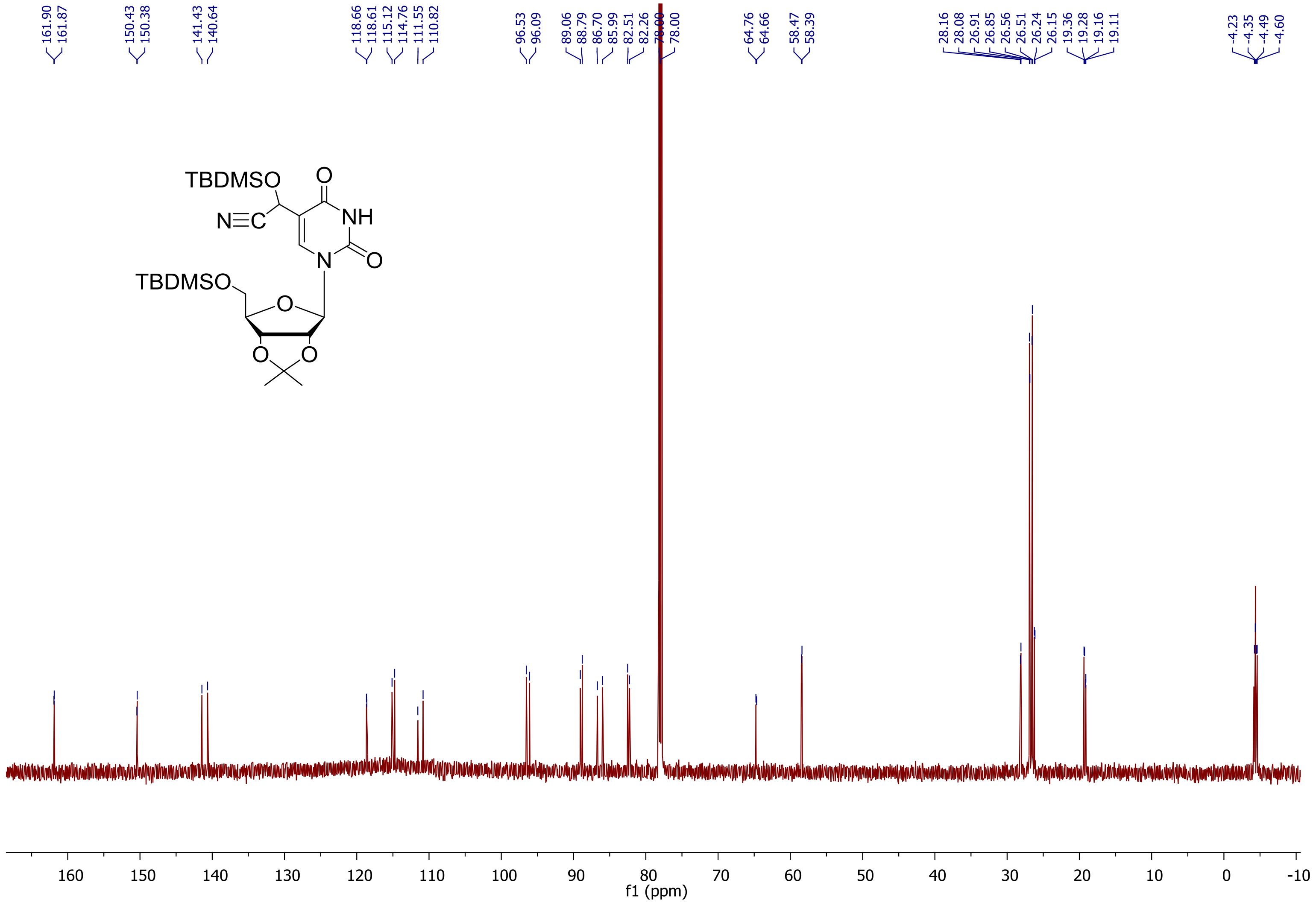

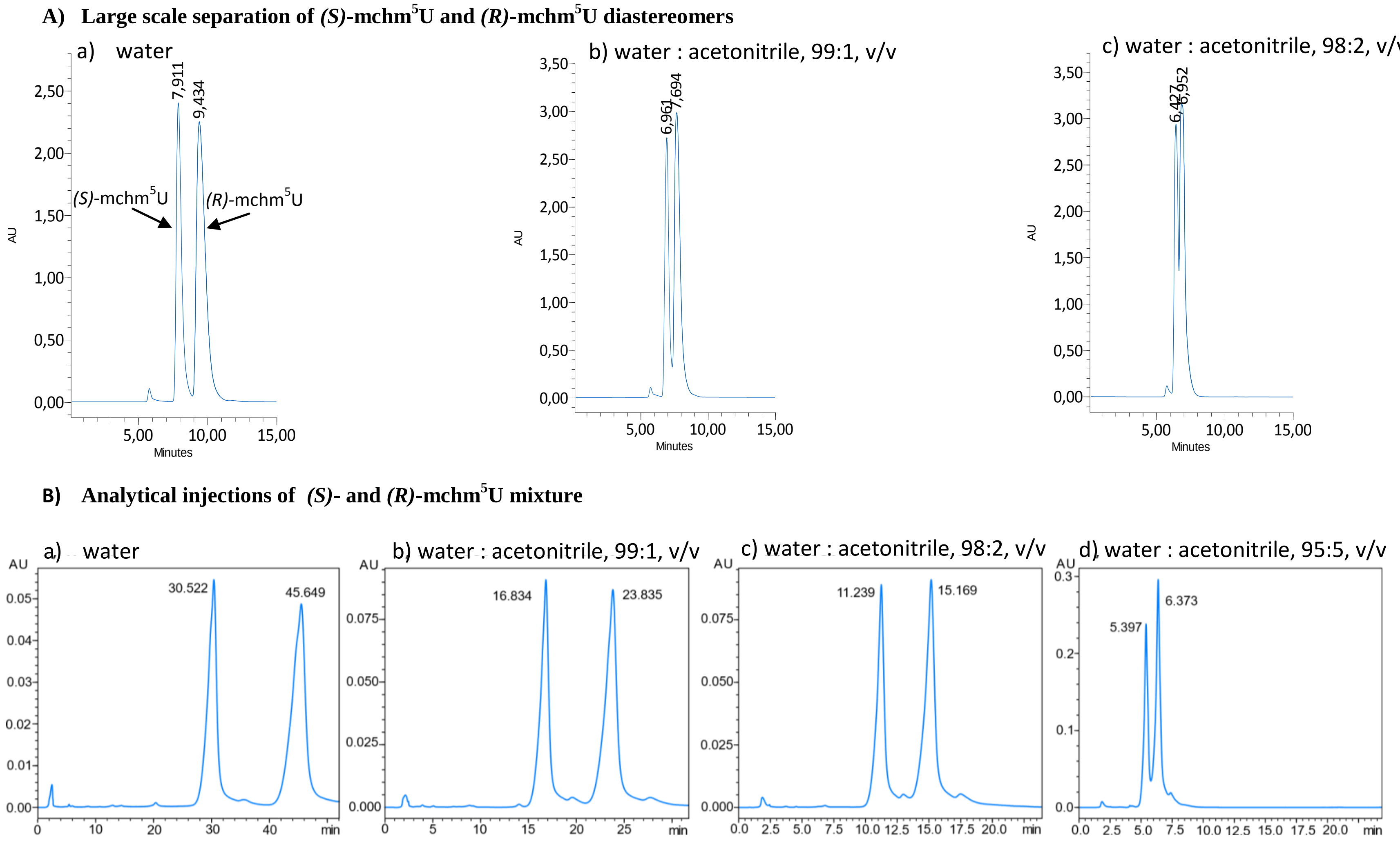

Figure S17. A) Large scale separation of *(S)*-mchm5U and *(R)*-mchm5U using isocratic RP-HPLC equipped with preparative C18 column (SUPELCO; Ascentis®; 25 cm/21.2 mm; 10 µm; flow 6 ml/min). Three types of eluents were tested: a) water; b) water:acetonitrile, 99:1, v/v; c) water:acetonitrile, 98:2, v/v; **B)** Analytical injections of *(S)* and *(R)* diastereomers mixture on Reprosher 100 C18 column (5µm 250x 4,6mm, flow 1 ml/min) to select conditions for separation of *(S)*-mchm5U and *(R)*-mchm5U on a large scale.

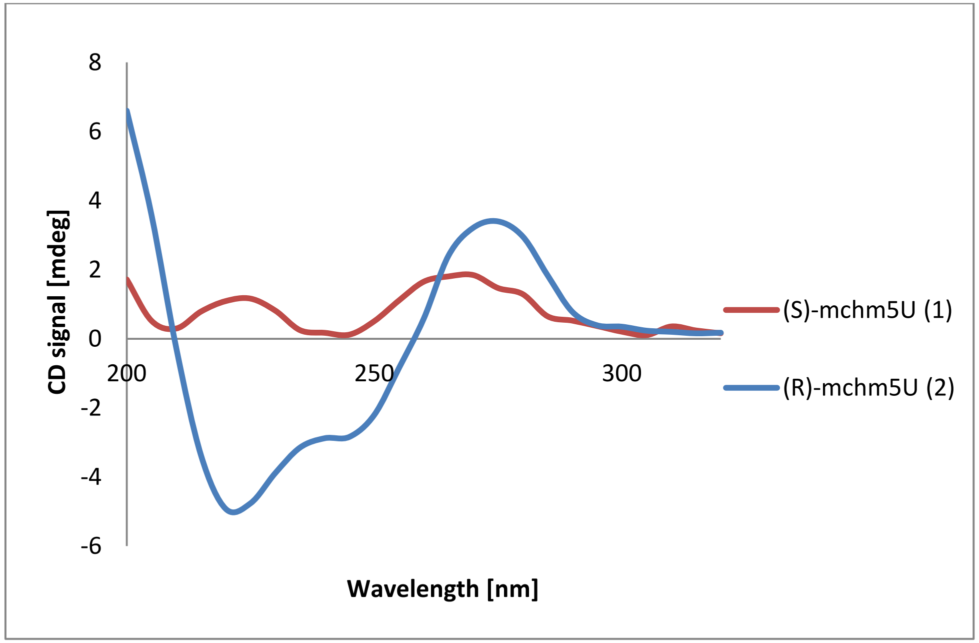

Figure S18. CD spectra of *(S)*-mchm^{5}U (**1**, red line) and *(R)*-mchm^{5}U (**2**, blue line).
